# Aphasia with Anomia in Mandarin Chinese: An Exploratory Pilot Study Based on Phonological Awareness Therapeutic Strategies

**DOI:** 10.3390/bs16101858

**Published:** 2026-10-09

**Authors:** Jiaying Chen, Yue Wang, Ying Li, Jinghui Huang, Fanfu Fang, Qin Wan

**Affiliations:** 1 Department of Rehabilitation Sciences, Faculty of Education, East China Normal University, Shanghai 200062, China; cjyrehab@163.com (J.C.); 51214109008@stu.ecnu.edu.cn (Y.W.); 2 Department of Rehabilitation Medicine, The First Affiliated Hospital of Naval Medical University, Shanghai 200433, China; yingli2653@foxmail.com (Y.L.); huangran_1234@163.com (J.H.); 3 Child Health and Rehabilitation Clinic, Department of Pediatrics, Qilu Hospital of Shandong University, Jinan 250014, China

**Keywords:** stroke, aphasia, anomia, mandarin-speaking, phonological awareness

## Abstract

Anomia, a pivotal symptom of aphasia, impedes functional communication. In tonal languages such as Mandarin, phonological awareness plays a critical role in lexical retrieval. This study developed and evaluated a phonological awareness-based intervention for anomia in Mandarin-speaking people with aphasia (PWA). Ten post-stroke Mandarin-speaking PWA with left-hemisphere damage and anomia (5M/5F; 4 non-fluent, 6 fluent aphasia) received a three-stage phonological awareness intervention (phoneme–nonword–real word). Pre- and post-intervention assessments covered naming ability, phonological awareness, and initial consonant articulation. After treatment, phonological awareness improved significantly across all measures (adjusted *p* < 0.05; partial η^2^ = 0.733–0.932). Both groups showed significant pre-to-post improvements in rhyme, alliteration, tone, phoneme awareness, syllable decoding, and syllable plausibility judgments (all adjusted *p* < 0.05); syllable awareness improved significantly only in the FA group (MD = 41.67, 95% CI = 24.85–58.48, adjusted *p* = 0.002). For naming accuracy, both groups improved significantly on trained words (FA: MD = 25.76, 95% CI = 13.08–38.44, adjusted *p* = 0.006; NFA: MD = 38.64, 95% CI = 29.30–47.98, adjusted *p* = 0.002). On the standardised test and untrained generalization words, only the FA group improved significantly (MD = 8.33, 95% CI = 4.05–12.62, adjusted *p* = 0.008; and MD = 11.37, 95% CI = 6.36–16.37, adjusted *p* = 0.004, respectively). Initial consonant articulation accuracy improved significantly in both groups (FA: MD = 17.46, 95% CI = 7.14–27.78, adjusted *p* = 0.014; NFA: MD = 25.00, 95% CI = 8.20–41.80, adjusted *p* = 0.036). Regarding naming errors, NFA showed significantly higher pre-treatment omission errors (median = 20.00% vs. 6.67%, adjusted *p* = 0.010) and phonological errors (median = 11.67% vs. 3.33%, adjusted *p* = 0.020) than FA on the standardized test; on trained words, NFA had higher pre-treatment omission errors (median = 15.91% vs. 6.82%, adjusted *p* = 0.048), while FA had higher post-treatment word formation errors (median = 9.09% vs. 0.00%, adjusted *p* = 0.038). No significant treatment-related changes in the occurrence rate of naming errors were detected on untrained generalization words. In conclusion, this exploratory pilot study provides preliminary evidence that a phonological awareness-based intervention may improve phonological awareness and trained-word naming ability in Mandarin-speaking PWA. Improvements on untrained words were limited and observed only in the FA group. Therefore, the intervention’s generalization remains uncertain. These findings cautiously support a language-specific rehabilitation approach attuned to Mandarin phonology.

## 1. Introduction

Aphasia is an acquired language disorder caused by organic brain damage, such as stroke, that results in the loss or impairment of previously acquired language functions (Savvidou et al., 2026). It is a prevalent clinical manifestation after stroke. The Boston diagnostic classification of aphasia categorizes aphasia into fluent aphasia (FA) and non-fluent aphasia (NFA) according to the fluency of spontaneous speech (Fong et al., 2019).

Given the heterogeneity of the causative lesions, aphasia can present as multiple forms of language dysfunction, encompassing diverse aspects of language comprehension and expression, including listening, speaking, reading, and writing. Aphasia can affect patients’ daily communication and social participation to varying degrees (Baker et al., 2022). Among them, anomia, defined as the inability to retrieve previously learned words, is a core and persistent symptom in PWA (Nardo et al., 2017).

Assessing naming ability is a fundamental element in both clinical practice and scientific research on aphasia. The confrontation naming task is a classic paradigm for clinically quantifying word-naming ability in PWA (Kiran et al., 2018). The Chinese version of the Boston Naming Test (BNT-C) is widely administered in China (Y. Li et al., 2022). Typically, the number of correctly named items or naming accuracy is used as an index of naming ability. Further research has indicated that anomia in the confrontation naming task can manifest at different levels within the lexical access system, resulting in distinct types of naming errors. Investigating naming errors can provide detailed insights into changes within the word retrieval and processing system (Kendall et al., 2013). In confrontation naming tasks, Mandarin speakers typically employ complete syllables with tones as the basic phonological encoding units. Their phonological processing is more holistic and rhythmically coherent. As a result, phonological paraphasias produced by Mandarin-speaking PWAs during naming predominantly manifest as errors within a preserved syllable framework, where the syllable structure itself (e.g., the integrity of initial consonants and final vowels) remains relatively stable. Because the association between orthographic form and semantic meaning is stronger than between orthographic form and phonological representation in Mandarin, semantic errors are also more prevalent. Furthermore, naming ability varies across aphasia types. FA demonstrates a significantly higher overall naming accuracy than NFA (Qian et al., 2022). In FA, naming errors primarily consist of semantic-related errors, whereas in NFA, the main error types include phonological-related errors and omissions.

Lexical access models, such as the Logogen Model, Levelt’s Model of Speech Production, and the Interactive Activation Model (IA model), all indicate that successful word retrieval necessitates processing at both semantic and phonological levels (Sayers et al., 2023). Consequently, behavioural therapies based on semantics, phonology, or both are the mainstream approaches for treating anomia in clinical practice. These treatments typically focus on training with a limited set of real words, resulting in limited efficiency in managing anomia clinically (Law et al., 2006; Kristinsson et al., 2021; Van Hees et al., 2013). Further research reveals that traditional semantic-based treatments tend to activate a broader lexical retrieval network. Still, this effect is largely confined to items that share many semantic features (e.g., body parts, foods, and clothing) (Kendall et al., 2019). Additionally, semantic networks correlate strongly with patients’ cognitive abilities and show substantial individual variability, making it difficult to develop systematic treatment protocols and impeding clinical implementation. In contrast, traditional phonological-based treatments generally do not aim to address the underlying problems within the speech production system itself. Instead, they leverage patients’ residual phonological capabilities to facilitate word retrieval. These treatments focus solely on the phonological decomposition and analysis of target words, without engaging the neural mechanisms underlying the intrinsic generalization of word retrieval. Consequently, their potential for generalization tends to be significantly inferior compared to semantic-based approaches.

Because Mandarin operates with a finite set of phonemes and phonotactic rules, relearning these rules is expected to enhance the processing capacity of the entire lexical retrieval network more efficiently. These rules represent phonological awareness, which can be defined as the awareness of arbitrary phonological units, such as syllables and intra-syllabic elements, and serves as a fundamental ability in word processing and retrieval. PWAs who experience anomia exhibit impairments across various aspects of phonological awareness tasks (Beeson et al., 2022; Howard & Gatehouse, 2006).

Awareness of phonemes is the most refined and critical sub-skill within the domain of phonological awareness. PWAs exhibit deficits in processing phoneme discrimination, phoneme identification, and restoring and integrating phonemic information into stable phonological representations (Marslen-Wilson & Warren, 1994). In Mandarin, syllables are typically composed of vowel and consonant phonemes.

The consonant phoneme at the onset of a syllable is often called the initial consonant, and awareness of the initial consonant within a syllable is termed awareness of alliteration (B. J. Tong, 2014). Currently, research on awareness of alliteration in Mandarin-speaking PWAs remains limited. However, alliteration cues are a commonly used clinical strategy in anomia interventions (Linebaugh et al., 2005). The therapeutic use of alliteration cues indirectly suggests that PWAs have difficulty extracting phonological representations associated with the alliteration of target words. Furthermore, impairments in alliteration extraction should be manifested in performance on alliteration awareness tasks.

In contrast to awareness of alliteration, both domestic and international scholars have conducted more comprehensive research on the awareness of rhyme. Assessments of rhyme awareness often use a rhyme discrimination task. This task can delineate the complete phonetic system and identify the brain regions actively involved in phonological retrieval among PWAs. It is commonly used diagnostically as an indicator of phonological function in post-stroke aphasia (Yen et al., 2019). A Japanese researcher analyzed rhyme discrimination tasks performed by 13 PWAs and found that accuracy of rhyme discrimination correlated with aphasia severity (Tanemura, 2006).

Mandarin syllables also include tones, which serve to distinguish meaning (Meckelborg et al., 2024). Learners of Mandarin process tone as a linguistic unit. Tones are closely associated with vowels within Mandarin syllables, and this association is particularly prominent in Pinyin (Y. Tong et al., 2008). Researchers often evaluate tone awareness through tone identification tasks. Mandarin-speaking PWAs commonly exhibit impairments in both tone perception and production (Yiu & Fok, 2009). Tone deficits can also be prevalent across various language tasks, including confrontation naming, oral reading, and reading comprehension (J. Wang, 2004).

Beyond the aforementioned components of phonological awareness, syllables composed of initials, finals, and tones serve as vital organisational units in speech planning. In natural speech production, speakers do not generate phonemes one by one. Instead, speakers group phonemes into articulatory gestures corresponding to syllables and then organise them into words (Romani et al., 2011). Within the phonetic stream of Mandarin production, syllables serve as an organizational framework that is easier to detect and segment than individual phonemes. Most studies indicate that, regardless of phonemic complexity, more syllables lead to more phonological errors in PWAs (Ziegler, 2005). In syllable awareness tasks involving syllable addition, deletion, or substitution, performance declines as the number of syllables increases. Increasing the number of syllables may increase pronunciation complexity, requiring more speech motor planning and programming and consuming more cognitive resources, resulting in poorer task performance (Baldo et al., 2011).

Theoretically, training to enhance phonological awareness has been shown to benefit all higher-level language tasks, including naming tasks (Silkes et al., 2019). Phonological awareness-based anomia interventions represented by Phonomotor treatment (PMT) have been extensively studied in English (Bislick, 2020; Braun et al., 2025; Castro et al., 2022; Kendall et al., 2019; Minkina et al., 2019). However, systematic treatment for anomia based on phonological awareness in Mandarin remains limited. Clinically, phonological therapies with limited efficiency, such as traditional PCA, are commonly employed. PCA typically involves analyzing the target word by its first sound, first sound association, ending sound, rhyming words, number of syllables, and tone (Python et al., 2025). Although this therapy also begins with the phonological dimension to help PWAs retrieve target words, it solely focuses on words requiring training. It does not incorporate comprehensive phonological awareness tasks. When extracting phonological features, it often relies on prompting strategies such as repetition, which fail to fully activate the relevant representations involved in phonological processing. The therapeutic approach demonstrated by PMT holds significant referential value for formulating phonological awareness treatment for Mandarin-speaking PWAs. However, the Parallel Distributed Processing Model of Phonology (PDP)-based intervention strategies within PMT also differ in their application to Mandarin versus English (Nadeau, 2001). First, orthographies are closely related to phonology, whereas Mandarin, as a logographic script, lacks a direct correspondence between the two. Therefore, written phonetic notation symbols (e.g., Pinyin) are required to replace the orthographic representation modules in English. Second, to develop phonological awareness, it is essential to integrate awareness of tone, a characteristic unique to Mandarin, to support comprehensive phonological processing training for word retrieval.

Consequently, this single-group exploratory pilot study designed and evaluated a phonological awareness-based naming intervention tailored to Mandarin-speaking PWAs, to provide a design basis for future definitive randomized controlled trials. This exploratory pilot study aimed to assess the feasibility and acceptability of the intervention and research protocol, explore patients’ responses to the intervention’s core components, estimate variability in candidate outcome measures for future sample size calculations, and identify practical barriers to clinical implementation.

Based on theoretical considerations and prior evidence, we hypothesized that the intervention would improve phonological awareness and naming ability in Mandarin-speaking PWAs, with gains likely differing between FA and NFA. Specifically, given the more severe phonological-articulatory deficits, we expected NFA to show less consistent improvement, particularly on untrained items. All subgroup comparisons were planned as exploratory.

## 2. Materials and Methods

### 2.1. Participants

Ten post-stroke Mandarin-speaking individuals with left-hemisphere damage and anomia, with an average age of 53.90 ± 13.63 years and an average disease duration of 3.3 ± 2.41 months, were recruited from a rehabilitation hospital in Shanghai between November 2023 and January 2024 through poster advertisements and information sessions. Eligible participants who volunteered were enrolled consecutively.

The inclusion criteria were as follows. Participants were diagnosed with left-hemisphere stroke, which was confirmed by CT or MRI. They were diagnosed with aphasia based on the Western Aphasia Battery. Anomia was confirmed by the BNT-C. Initial consonant articulation deficits were identified through the Chinese Articulation Assessment (Ge et al., 2024). Impairments in phonological awareness were determined by the Phonological Awareness Assessment Tool for Mandarin-speaking PWAs (PAAT) (Chen et al., 2026, online-first).

Additionally, participants were required to be able to repeat the non-meaningful syllable /pataka/ with cueing. Their vital signs needed to be stable. The minimum post-stroke duration was one month. Participants needed normal mental status, vision, and hearing. Basic auditory comprehension abilities were essential. The minimum educational attainment was junior high school. Mandarin was the primary language, and participants also needed basic Pinyin proficiency.

All enrolled participants received routine synchronous care not specifically targeted at linguistic function (e.g., general medical management, occupational therapy, and physical therapy, etc.).

The exclusion criteria were as follows. Participants were excluded if they scored 20 points or below on the Mini-Mental State Examination (MMSE). The MMSE cut-off was informed by observations from our preliminary pilot work, in which participants scoring at or below 20 often had difficulty following multi-step naming instructions, completing treatment sessions, and providing reliable data. We therefore used this threshold as a pragmatic operational criterion to exclude individuals with severe global cognitive impairment that could compromise intervention fidelity and assessment validity. The presence of disorders of consciousness or psychiatric conditions led to non-cooperation. Other exclusionary factors were severe apraxia of speech, oro-facial apraxia, autotopagnosia, or prosopagnosia. A comorbid diagnosis of a degenerative neurological disease, such as Alzheimer’s disease or Parkinson’s disease, also led to exclusion. The demographic and basic clinical characteristics available from participants’ medical records are presented in Table 1. Standardized quantitative data on aphasia severity and comorbid conditions were not available and are not reported.

This study was approved by the Institutional Review Board of the collaborating hospital and East China Normal University [ECNU Medical Ethics Review No. (2024) No. (8)]. Appropriate informed consent and details were provided to all participants before the study.

### 2.2. Treatment Program

#### 2.2.1. Therapeutic Strategy

PMT is a phonological awareness-based intervention for anomia that has been validated in the English context through multiple clinical studies. It aims to promote and strengthen phonological representations as well as phonological production and perception, thus improving the efficacy and accuracy of naming. The present study adapted the intervention logic and framework of PMT to develop a phonological awareness-based strategy for anomia within the Mandarin context. The specific treatment principles were as follows.

##### Emphasize Phonological Awareness-Based Tasks

The phonological awareness-based naming intervention did not employ the traditional confrontation naming approach. Instead, it emphasized phonological awareness-based training tasks. In terms of format, the phonological awareness treatment for oral naming integrated both perception and production tasks. In other words, the phonological awareness production treatment was implemented based on phonological awareness perception tasks.

In terms of complexity, treatments were classified into simpler detection tasks and more challenging manipulation tasks. Detection tasks included modalities such as identification and judgment. Manipulation tasks included operations like segmentation, blending, addition, deletion, and substitution (Lerner & Lonigan, 2016; Yang et al., 2024). During treatment, the progression moved from easier to more difficult tasks, beginning with detection tasks before advancing to manipulation tasks.

##### Multi-Modal Phonological Representation Domain Based on the Revised PDP Model

This study adapted the PDP model for the Mandarin context. The adaptation involved modifying the original PDP model by replacing the orthographic domain with a domain of written Pinyin symbols. This replacement was necessary because Mandarin orthography refers to structural components such as character radicals, which lack a transparent grapheme-phoneme correspondence and thus could not directly serve as a phonological representation domain. In contrast, Pinyin functions as the phonetic annotation system for characters. It explicitly represents syllable components and is integral to phonological awareness development. Consequently, phonological awareness-based treatments were implemented through five modalities: auditory, articulatory-motor, tactile-proprioceptive, visual, and written Pinyin (Table 2).

The PDP model does not specify treatment priorities across different phonological representation domains. Research indicates that naming performance for PWAs is significantly better with visual stimulation than with auditory stimulation (Zhu, 2014). Therefore, the incorporation of phonological representation domains followed the following principles. Visual stimulation was prioritized over auditory stimulation for initial incorporation. Tactile-proprioceptive input was emphasized throughout treatment. Written Pinyin was introduced in later phases of therapy, tailored to each patient’s individual needs.

Furthermore, each phonological awareness task typically encompassed multiple representation domains. At the initial phase of treatment, a patient’s stronger domain was paired with a weaker one to reinforce various aspects of phonological awareness representation. For example, in the case of a patient capable of identifying and describing articulatory movements yet exhibiting a less robust response to auditory stimuli, the initial approach could involve using a mirror to observe and recognize the noted articulatory actions. Simultaneously, questions could be posed, and examples of the sounds produced by these movements could be provided. In general, a phonological awareness task that incorporated more representation domains was easier. As treatment progressed, the support from multiple domains was gradually reduced. The ultimate goal was for the patient to complete the phonological awareness task using the most challenging domain alone (e.g., the auditory domain in isolation).

##### Select Treatment Corpus from Easy to Difficult

To better minimize semantic influence and specifically enhance phonological awareness, this treatment was divided into three phases. Each phase corresponded to a distinct set of treatment materials. Participants could progress to the next phase when training accuracy in the current phase exceeded 80%.

The first phase focused on training the segmental and suprasegmental phonemes that constitute words. It aimed to access the multi-modal representations of phonology and enhance the ability to relearn phonemes. The optimal treatment outcome is reconstructing the multi-modal distributed representations within each phoneme. This means that any phonological representation provided for a specific phoneme will instantly generate its corresponding distributed representation. For instance, presenting the phoneme /b/ in auditory form (the therapist says /b/ to the patient) should immediately activate the distributed visual, written Pinyin, articulatory-motor, and tactile-proprioceptive representations of /b/. Because each phoneme shares identical distributed phonological representation domains, a robustly established representation pattern can generalize to all phonemes in Mandarin. Thus, the established pattern allows the patient to cue themselves during treatment.

The second phase focused on training with nonwords. It was designed to specifically train phonology in the absence of concepts and semantics. Since the outcomes of phonological awareness manipulation tasks cannot be predicted, the accidental creation of real words (e.g., a tone substitution task: qiǒng → qióng) cannot be entirely avoided. Nevertheless, during treatment, the therapist must ensure that the primary emphasis remains on phonology rather than semantics. The optimal outcome of treatment is that patients can detect and manipulate phonological features within the provided nonword. Moreover, when confronted with challenges or errors during these tasks, the patient should be able to leverage the distributed phonological representation domains established in the first phase for self-cuing and self-correction.

The third phase focused on training with real words to maximize the naming ability of functional words. Consistent with the nonwords in the second phase, during phonological awareness tasks involving manipulation, word attributes alternate between real words and nonwords. Regardless of whether the words convey actual meaning, the core of the treatment remains the phonological awareness-based task. The optimal outcome is that patients can detect and manipulate phonological features within the provided real words. Moreover, when confronted with challenges or errors during these tasks, the patient should be able to leverage the distributed phonological representation domains established in the first phase for self-cuing and self-correction.

##### Guided Questioning Strategies

During treatment, the therapist provided cues and guided the session through exploratory and analytical questions. Traditional repetition-based naming allows patients to directly copy or activate the target phonological form without undergoing internal association or the process of phonological access. Consequently, they typically lack a generalization effect. Typically, word-naming generalization improves when therapists provide less direct assistance and patients use more self-directed strategies (Francis et al., 2002). Therefore, this study employed a guided questioning strategy. This strategy encouraged patients to independently explain, analyze, and recall relevant phonological representation domains. It also guided them to identify and understand problems that arise during phonological awareness tasks, and to evaluate and correct their own errors. The therapist did not simply provide the correct answer to tasks. Table 3 presents examples of guided questions used in the treatment.

#### 2.2.2. Treatment Stimulus

As shown in Table 4, the stimuli include 4 minimal consonant phoneme pairs, 3 core vowels, 4 tones carried by these 3 core vowels, 17 nonwords, and 22 real words.

PWAs typically have significant difficulty perceiving and producing minimal phonemic pairs (Martin et al., 2025). Detailed phonological differences require more cognitive resources, thereby reinforcing phonological processing of words. Based on each patient’s assessment of initial consonant articulation, we selected 2 pairs of unimpaired minimal consonant phonemes and 2 pairs of impaired minimal consonant phonemes. The unimpaired pairs indicated that patients could successfully access the articulatory-motor domain of the phonological representation for these sounds. Training on these phonemes facilitated transfer of representations across domains, helping establish a complete phonological representation network. Once patients had completed training on the unimpaired pairs, they were able to generalize their acquired knowledge to the impaired phonemes. Because vowels are more abstract within the phonological representation system than consonants, they are more challenging for patients to comprehend. Therefore, we selected three core Mandarin vowels: /a/, /i/, and /u/. Meanwhile, we used /a/, /i/, and /u/ as carriers to train the four tones.

Nonwords stimuli were classified into three categories based on their morphological similarity to real words: high word-likeness nonwords, low word-likeness nonwords with nonexistent tone combinations, and low word-likeness nonwords with nonexistent phonotactic combinations. High word-likeness nonwords are meaningless words composed of syllables that exist in Mandarin (e.g., mēn děi) (Jiang, 2017). Low word-likeness nonwords are meaningless stimuli composed of phonotactically illegal syllables in Mandarin and can be categorized as nonwords with nonexistent tone combinations (e.g., béi) and nonwords with nonexistent phonotactic combinations (e.g., piā) (N. H. Li & Cheung, 2014).

Twenty-two real-word stimuli with 100% naming consistency were selected (Liang, 2023). The entire corpus provided 100% coverage of initial consonants, 84.62% coverage of final vowels, 100% coverage of monosyllabic tones, and 75% coverage of disyllabic tone combinations.

The real words consist of 22 items with 100% naming consistency (Martin et al., 2025). The stimulus set included all 23 Mandarin consonants (100% coverage) and 33 of the 39 Mandarin vowels (84.62% coverage). It also incorporated all monosyllabic tones (100% coverage) and 75% of the disyllabic tone combinations.

#### 2.2.3. Treatment Protocol

**Figure 1:**
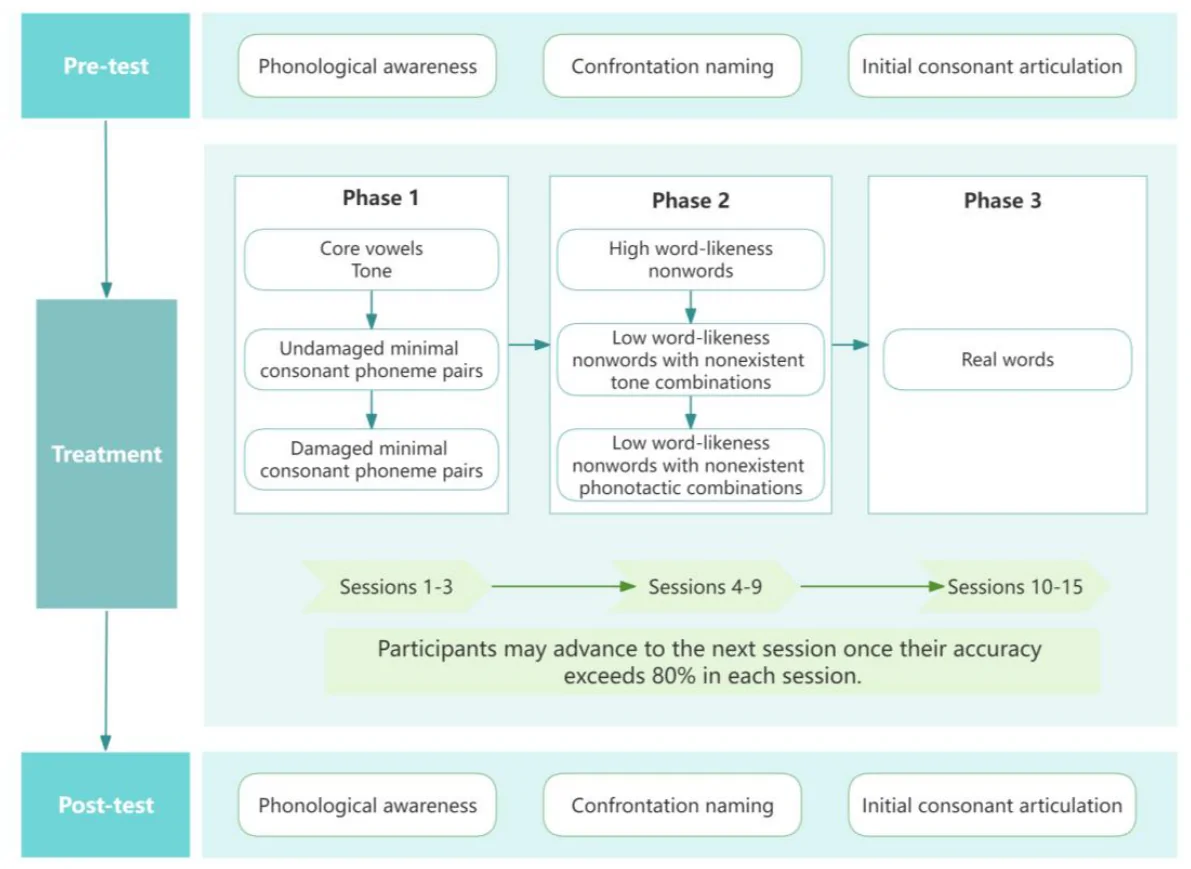
The treatment protocol includes a pre-test, three treatment phases, and a post-test. Phase 1 includes three phoneme-based training sessions, while Phase 2 and Phase 3 each consist of six training sessions, respectively based on nonwords and real words.

**Figure 2:**
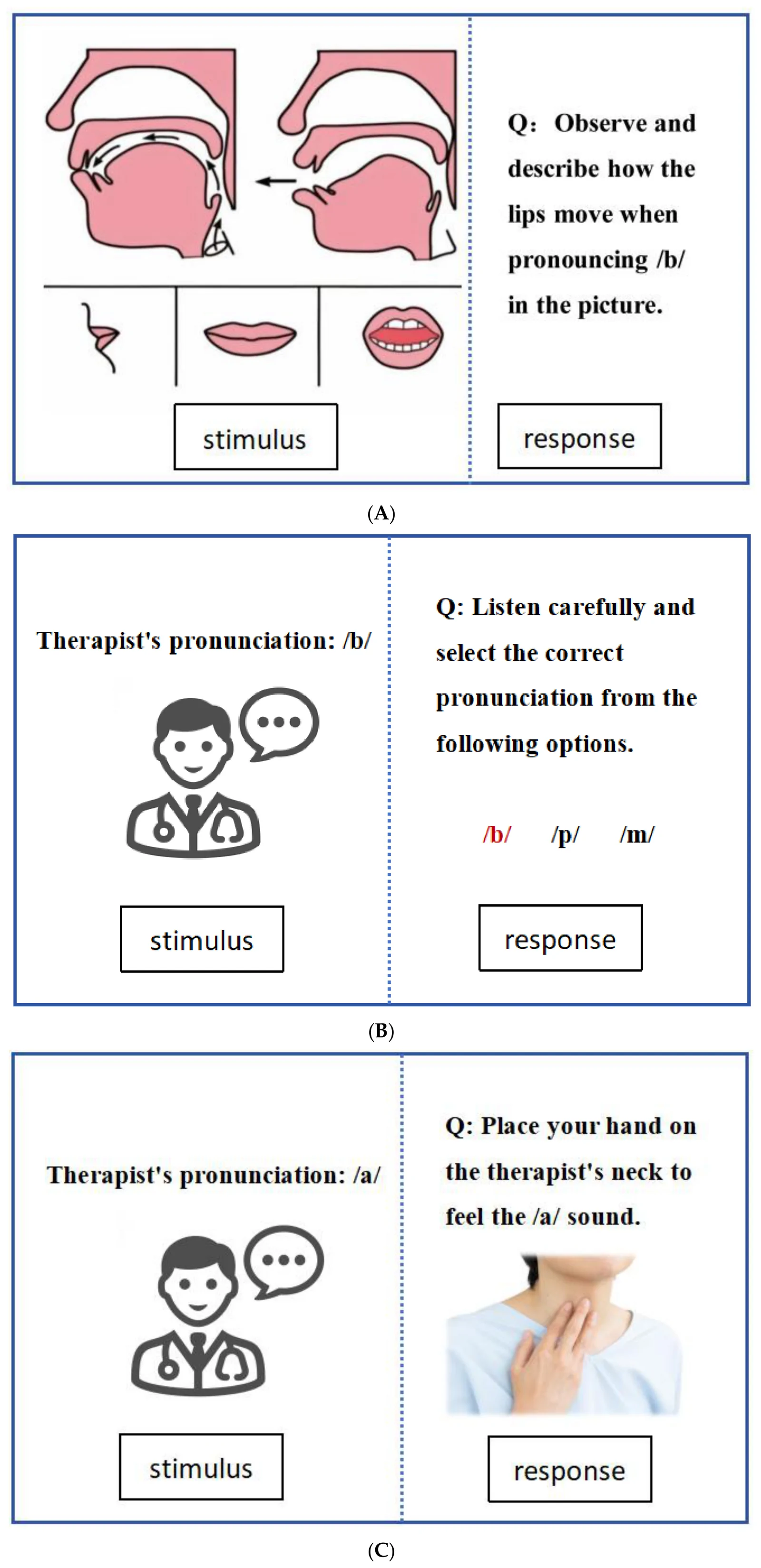

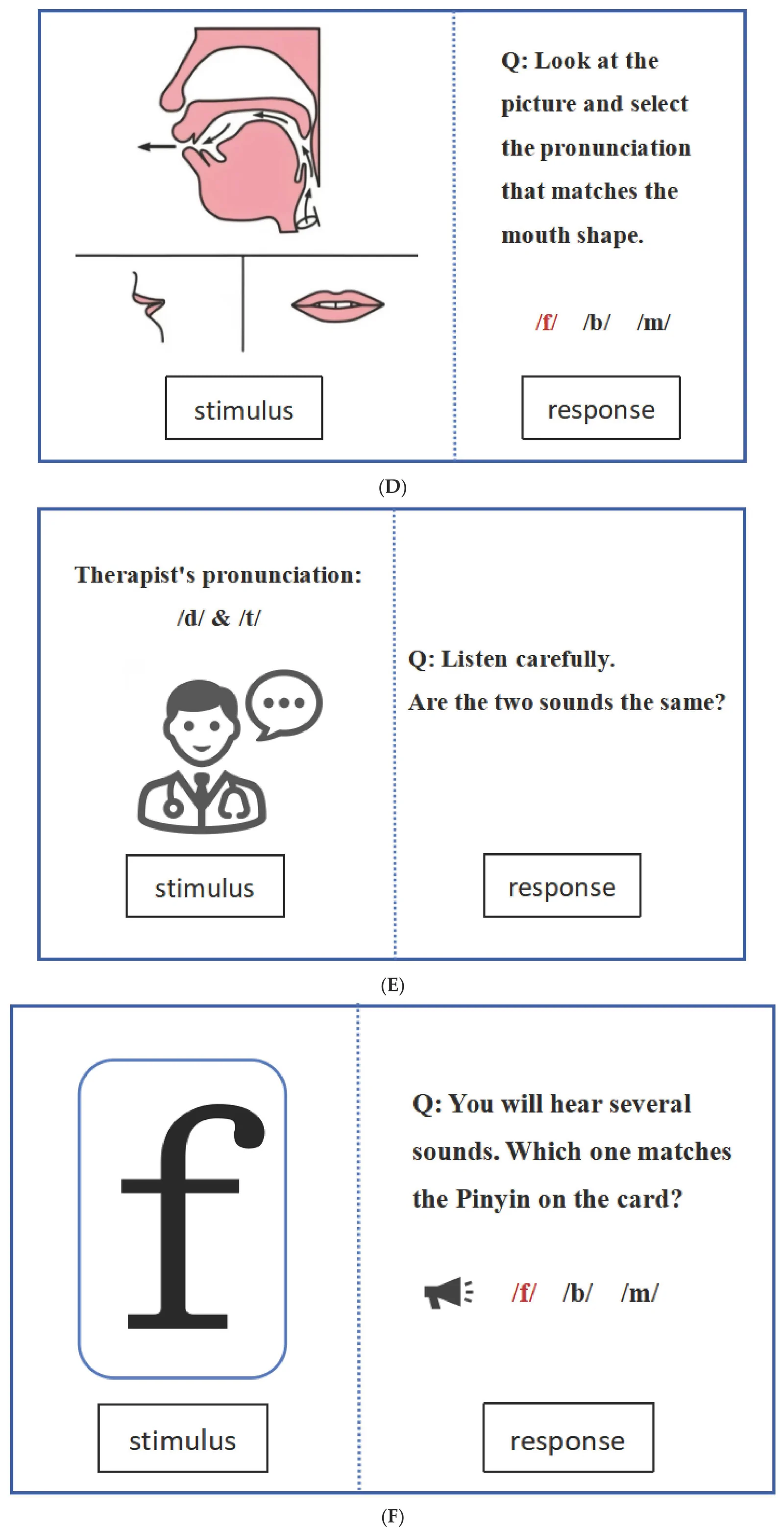
Examples of phonological awareness perceptual training based on multi-modal phonological representations for phonemes. (**A**) Describing lip movement when pronouncing /b/; (**B**) Producing /b/ and asking the patient what they hear; (**C**) Placing a hand on the neck while pronouncing/a/to feel whether the vocal cords are vibrating; (**D**) Presenting the mouth shape for/f/and asking the patient to identify the corresponding phoneme; (**E**) Determining whether /d/ and /t/ represent the same phoneme; (**F**) Presenting a Pinyin card for /f/ and asking the patient to match it from among several speech sounds.

The treatment protocol consisted of 15 sessions, each lasting one hour, administered once daily, five days per week, over three weeks. The treatment was conducted by a professional speech therapist. As described previously, this therapy was divided into three phases, as illustrated in Figure 1. Specifically, Phase 1 comprised Sessions 1–3, Phase 2 comprised Sessions 4–9, and Phase 3 comprised Sessions 10–15. Participants advanced to the next session once their accuracy exceeded 80% in each session. The number of repetitions or trials per task was not fixed. Instead, the therapist individualized it based on each participant’s performance, attention, and fatigue level, in order to provide adequate therapeutic stimulation while minimizing frustration and exhaustion. No self-administered therapy or home-based practice was assigned, as the intervention required professional guidance from a qualified speech therapist. The specific treatment protocol was as follows:

The first phase aimed to train phonemes through a multi-modal phonological representation domain, guiding patients to relearn the auditory, visual, articulatory-motor, tactile-proprioceptive, and Pinyin representations of phonemes. Training began with detection tasks targeting minimal consonant phoneme pairs, core vowels, and tones, encompassing both perception and production training. The perceptual training in phonological awareness primarily included the following paradigms: visual description (e.g., describing lip movement when pronouncing /b/, Figure 2A); auditory description (e.g., producing /b/ and asking the patient what they hear, Figure 2B); tactile-proprioceptive description (e.g., placing a hand on the neck while pronouncing /a/ to feel whether the vocal cords are vibrating, Figure 2C); visual discrimination (e.g., presenting the mouth shape for /f/ and asking the patient to identify the corresponding phoneme, Figure 2D); auditory discrimination (e.g., determining whether /d/ and /t/ represent the same phoneme, Figure 2E); and Pinyin matching (e.g., presenting a Pinyin card for /f/ and asking the patient to match it from among several speech sounds, Figure 2F).

**Figure 3:**
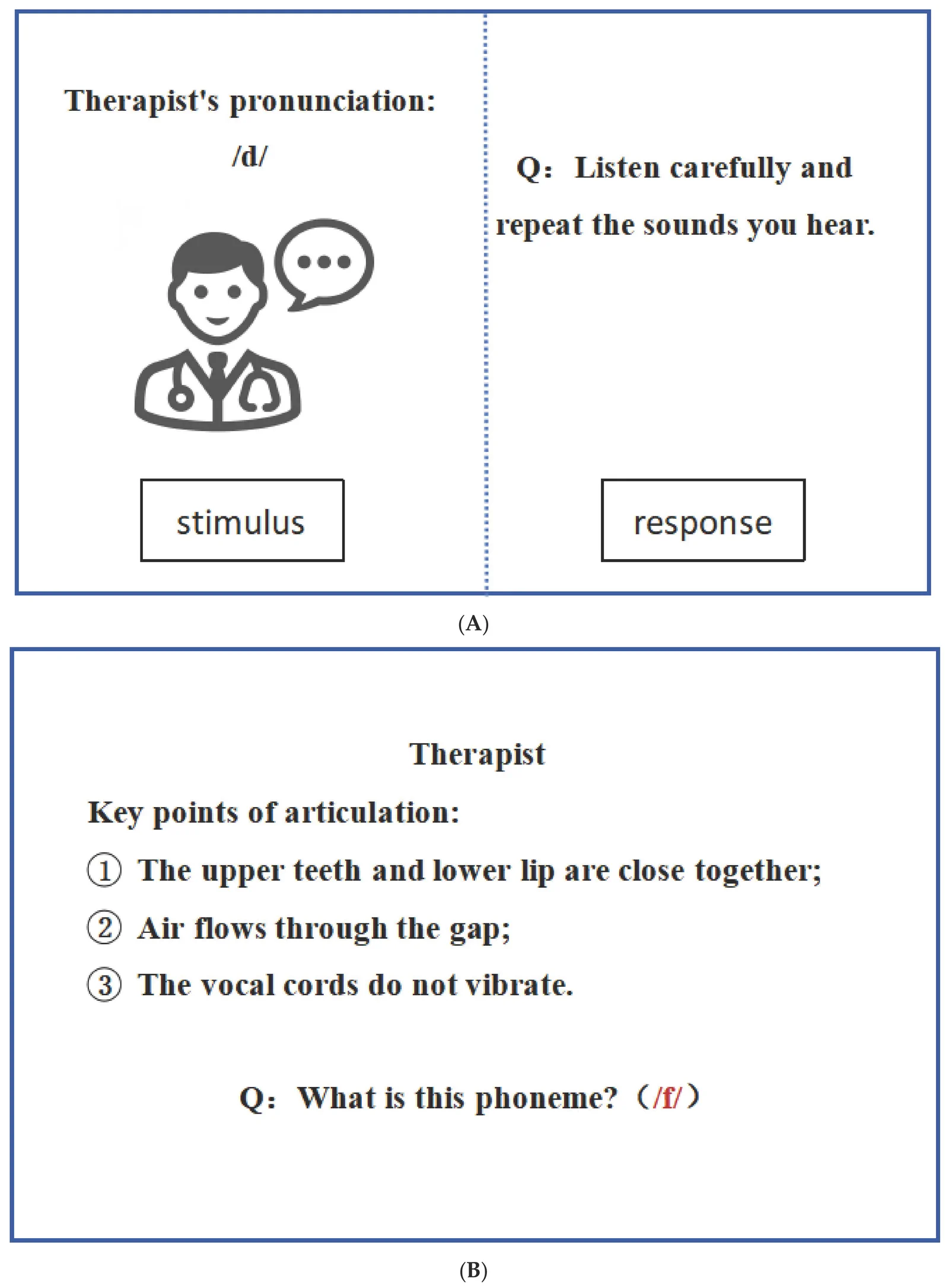
Examples of phonological awareness production training based on multi-modal phonological representations for phonemes. (**A**) Phoneme repetition; (**B**) Describing the articulation place and mode of /f/ and asking the patient to pronounce the phoneme; identifying the corresponding phoneme by observing the mouth shape; identifying the phoneme based on its Pinyin.

**Figure 4:**
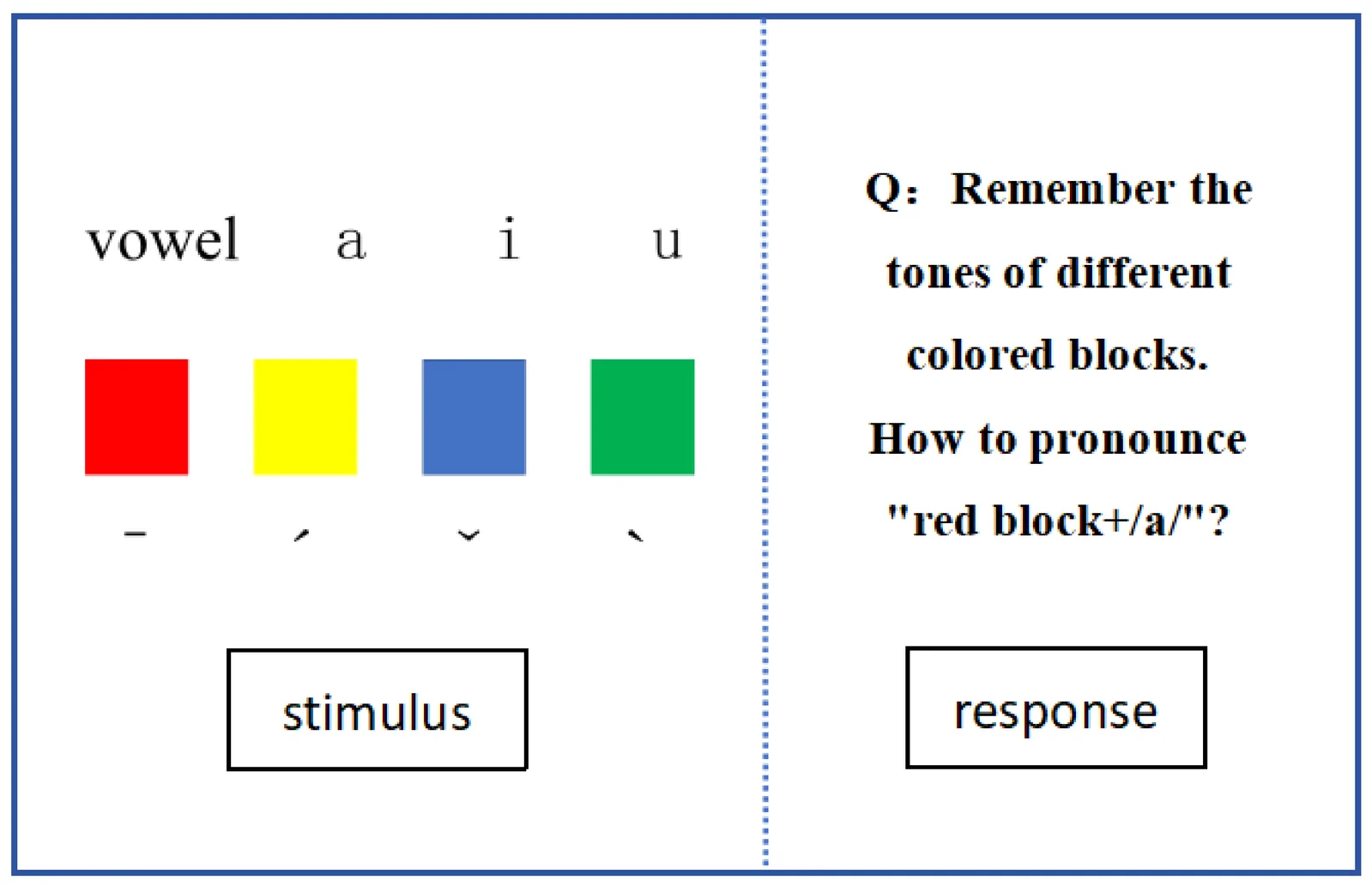
An example of phonological awareness manipulation training based on multi-modal phonological representations for phonemes.

The production training in phonological awareness mainly included the following paradigms: phoneme repetition (Figure 3A) and phoneme production (e.g., describing the articulation place and mode of /f/ and asking the patient to pronounce the phoneme; identifying the corresponding phoneme by observing the mouth shape; identifying the phoneme based on its Pinyin, Figure 3B). As performance improved, the therapist introduced colored blocks representing consonants, vowels, and tones for manipulation tasks. For example, red blocks represented “high level tone,” yellow blocks represented “rising tone,” blue blocks represented “falling-rising tone,” and green blocks represented “falling tone.” When the patient had memorized these representations, the therapist selected /a/ and paired it with a red block, asking the patient to pronounce /ā/ (Figure 4). When the overall treatment accuracy in the first phase surpassed 80%, the patient could advance to the subsequent phase of training; the same principle applied to the following phases.

The second and third phases of the therapy concentrated on phoneme sequences. Training used both real words and nonwords. The patient was instructed to spell words and concentrate on training awareness of alliteration, rhyme, syllables, tone, and phonemes. Simultaneously, the multi-modal phonological representation domains obtained in the first phase were employed as a self-cueing strategy to assist patients. The subsequent section presents a therapeutic case, using the nonword /béi/ as an exemplar.

First, the therapist presented the nonword /béi/ in written form and instructed patients to spell it independently using the skills acquired in phase one. If the patient made an error in the consonant, vowel, or tone, the therapist used guided questioning to prompt self-correction by drawing on the multi-modal representation domains learned earlier. For example, if the patient mispronounced /béi/ as /bēi/, the therapist would articulate both forms and ask whether they sounded identical. Through guided questioning, the therapist then helped the patient identify specific auditory differences between them.

**Figure 5:**
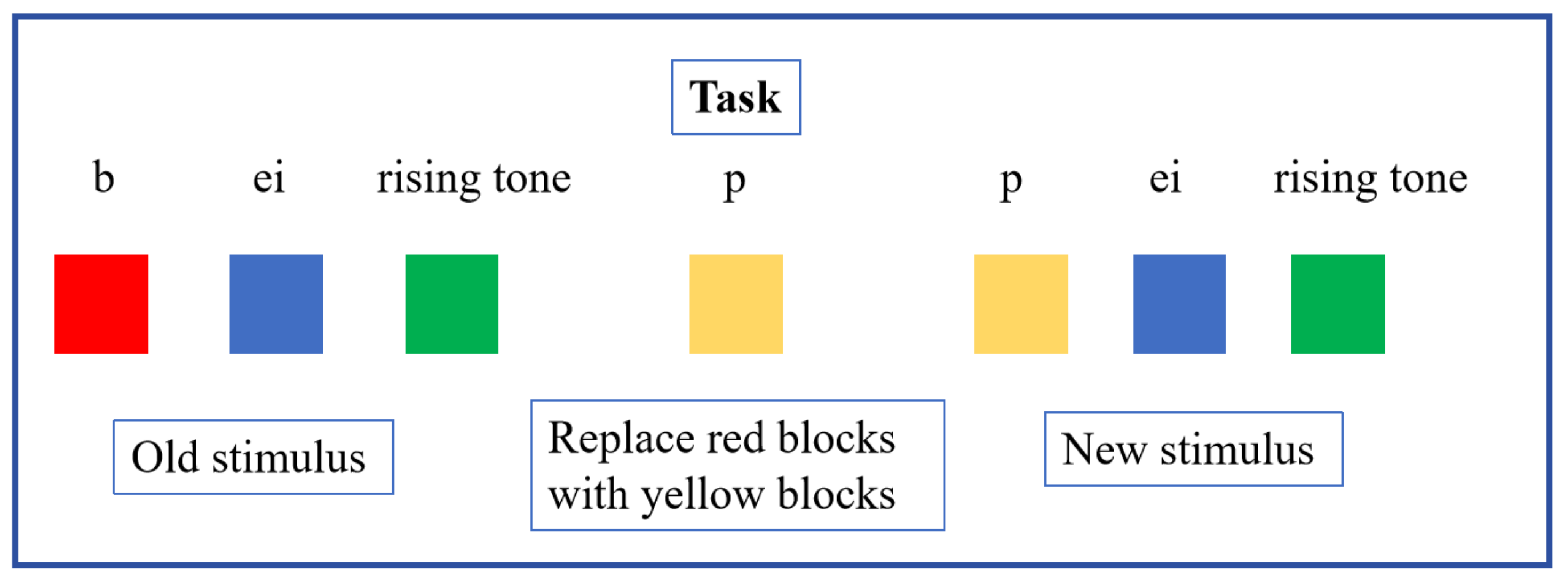
Example of alliteration awareness training: The initial consonant, final vowel, and tone of /bei/ were respectively represented by a red block, blue block, and green block. Subsequently, the therapist replaced the red block with a yellow block representing /p/ and instructed the patient to pronounce the modified sound.

Second, the therapist prompted the patient to describe the laryngeal sensation while producing the initial sound /b/. Subsequently, the therapist inquired about the movements of the articulatory organs for that sound. The patient was then asked to select a picture depicting the corresponding articulatory gesture and to identify the Pinyin symbol for the initial sound. Additionally, the patient used differently colored blocks to represent the phonological components of /béi/, completing phoneme segmentation and blending tasks. Finally, the therapist modified the blocks representing the initial of /béi/ (i.e., performing phoneme deletion, addition, or substitution tasks) and instructed the patient to produce the modified form (Figure 5).

**Figure 6:**
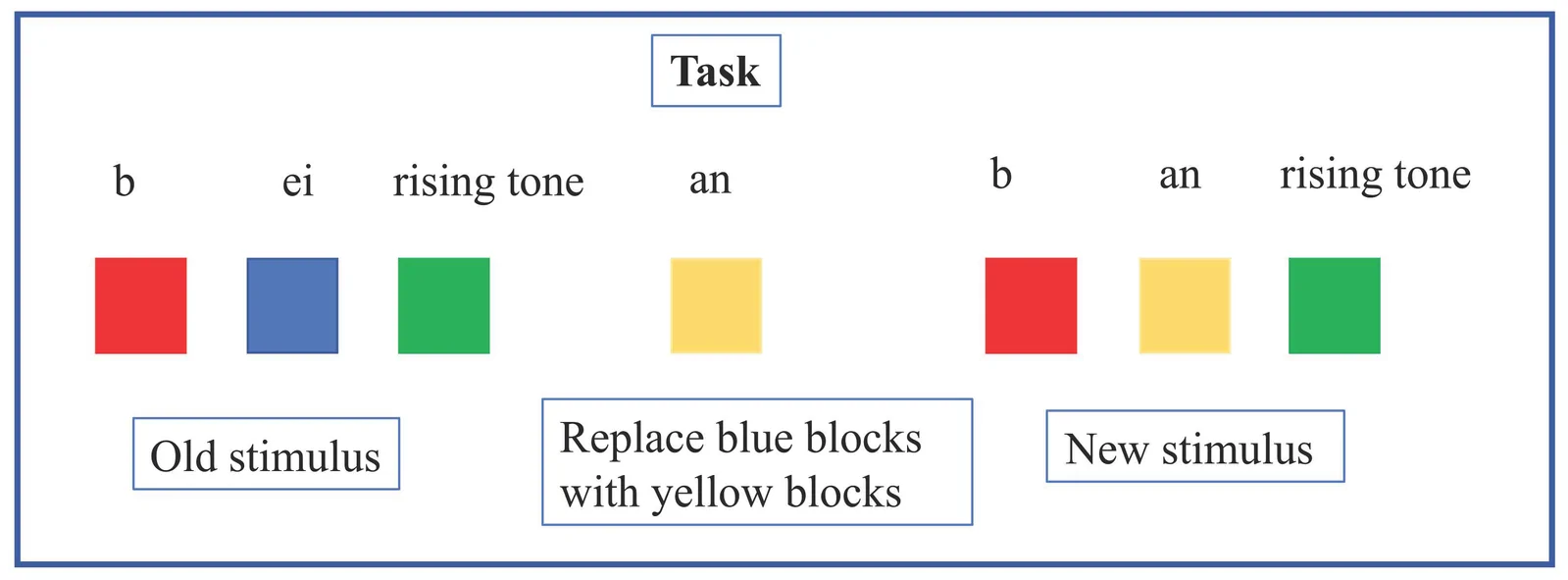
Example of rhyme awareness training: The initial consonant, final vowel, and tone of /bei/ were respectively represented by a red block, blue block, and green block. Subsequently, the therapist replaced the blue block with a yellow block representing /an/ and instructed the patient to pronounce the modified sound.

Third, the therapist guided the rhyme-focused phonological tasks as in step 2 (Figure 6).

**Figure 7:**
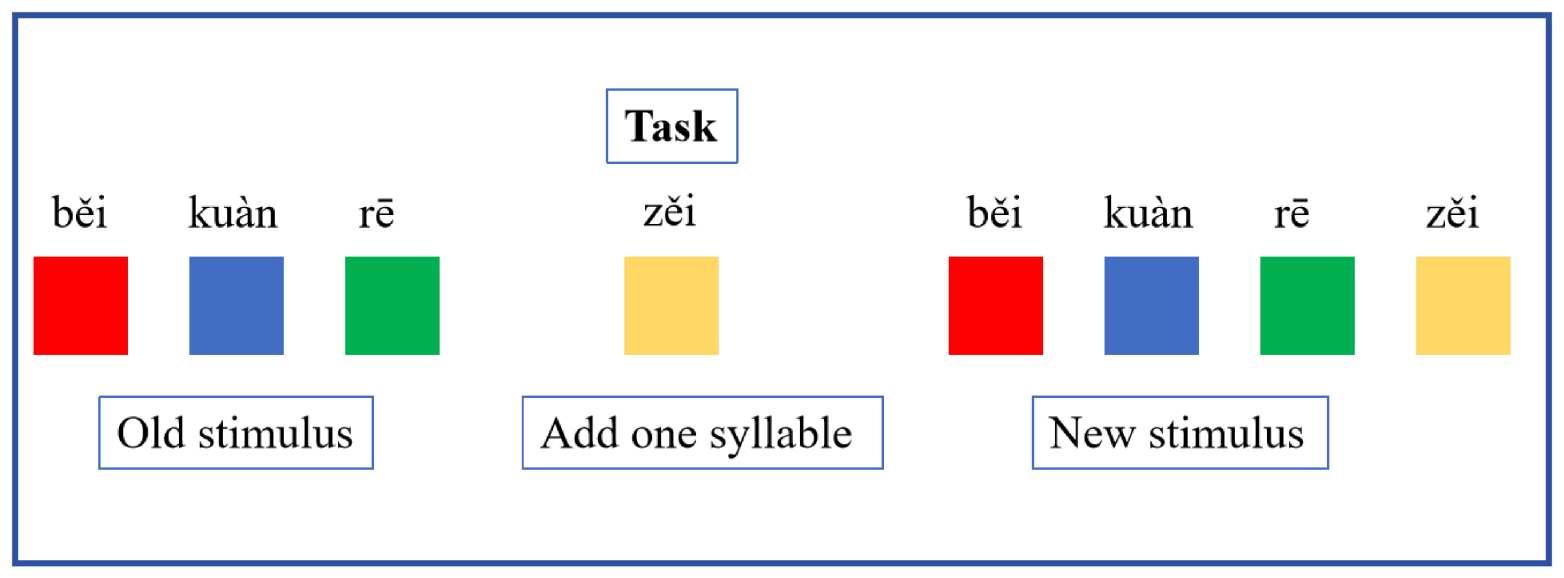
Example of syllables awareness training: Three nonwords were respectively represented by a red block, blue block, and green block. Subsequently, the therapist added a yellow block representing /zěi/ after the three syllables and instructed the patient to pronounce the modified sound.

Fourth, the therapist guided the patient to compare laryngeal sensations and the frequency/amplitude of articulatory movements while producing various syllables, including /béi/. The patient matched each syllable with the corresponding articulatory action pictures, Pinyin cards, and tone cards. The patient was also instructed to use colored blocks to represent different syllables that include /béi/. Finally, the therapist deleted, added, or substituted the colored blocks representing different syllables and asked the patient to produce the modified syllable combinations (Figure 7).

**Figure 8:**
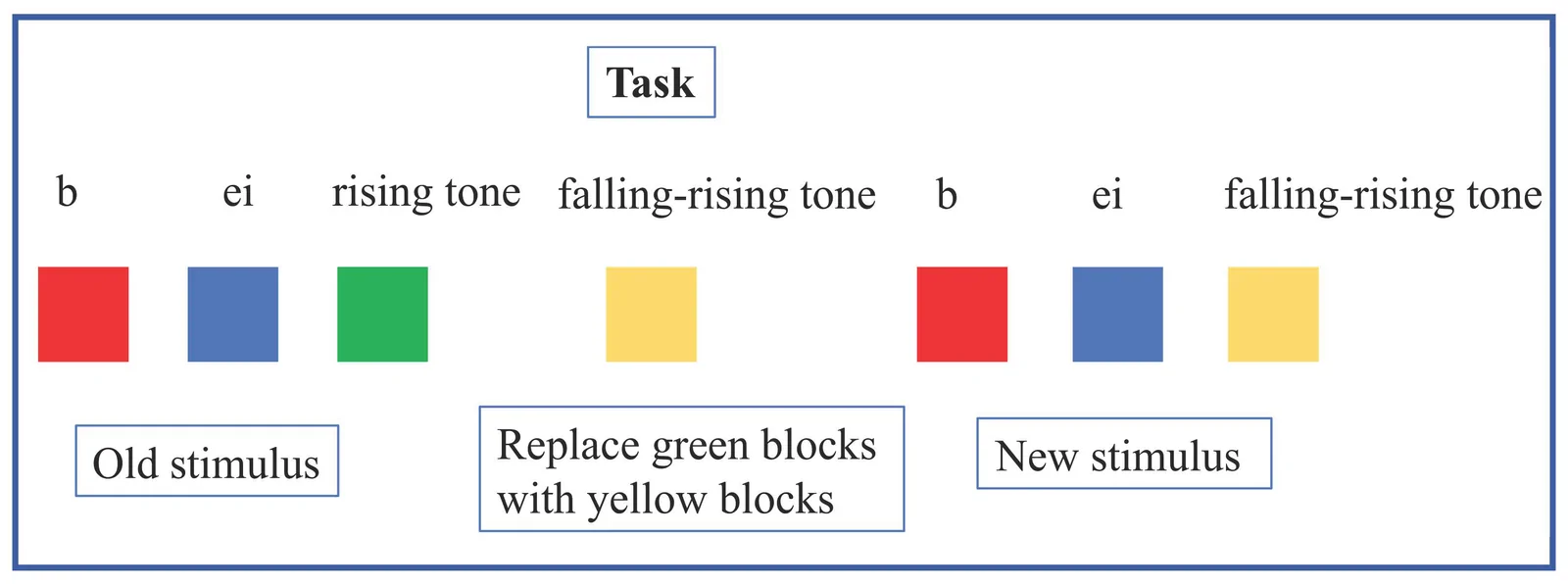
Example of tone awareness training: The initial consonant, final vowel, and tone of /bei/ were respectively represented by a red block, blue block, and green block. Subsequently, the therapist replaced the green block with a yellow block representing a falling-rising tone and instructed the patient to pronounce the modified sound.

Fifth, the therapist guided the patient to describe the laryngeal sensation, voicing duration, and pitch contour when producing the tone of /béi/. The therapist also presented the rising tone card and demonstrated the associated hand gesture. The therapist then asked the patient to represent different phonological components of /béi/ using different blocks. Finally, the therapist replaced the blocks corresponding to rising tone and asked the patient to produce the modified form (Figure 8).

Finally, although phonemic awareness was also incorporated into other phonological awareness tasks, phoneme awareness training placed additional emphasis on using colored blocks for phoneme blending tasks and judgments of syllable plausibility. Throughout the treatment, the therapist consistently used guided questioning to refine and consolidate the patient’s phonological awareness.

### 2.3. Outcome Measures

The outcome measures of this study mainly included phonological awareness characteristics, naming ability, and initial consonant articulation ability in Mandarin-speaking PWAs.

Phonological Awareness was assessed by the PAAT (Chen et al., 2026). PAAT consists of 75 items divided into five sections: awareness of rhyme, awareness of alliteration, awareness of syllables, awareness of tone, and awareness of phonemes. For each subsection, patients performed tasks based on auditory stimuli, including rhyme judgment, alliteration judgment, syllable deletion, tone judgment, phoneme blending, and judgment of syllable plausibility. The accuracy of phonological awareness was calculated by dividing the number of correct responses by the total number of responses and multiplying by 100%.

Naming ability was assessed in three sections: Assessment 1 utilized the BNT-C (Y. Li et al., 2022). The BNT-C consists of 30 black-and-white line drawings selected from the full BNT. It demonstrates good internal consistency (α = 0.738) and has been widely used in anomia research within the Mandarin context. Assessments 2 and 3 each consisted of 22 pictures of high-frequency nouns characterized by high naming consistency and a balanced distribution of consonants, vowels, and tones (Liang, 2023). Specifically, Assessment 2 served as the real-word stimuli used during treatment.

In contrast, Assessment 3 was employed to test the generalization effect of phonological treatment on naming ability and was not employed during the intervention phase. For each assessment, confrontation naming accuracy was calculated by dividing the number of correct responses by the total number of responses and multiplying by 100%. In addition, the types of naming errors patients made during assessment were recorded and analyzed based on the IA model in a Mandarin context (Qian et al., 2022).

The initial consonant articulation was assessed using the items in the initial consonants section of the Chinese Articulation Assessment (Ge et al., 2024). The assessment employed a repetition format to evaluate the accuracy of initial consonant production. To ensure reliability, participants repeated each item three times. The assessor recorded the number of items produced correctly and then converted them into initial consonant articulation accuracy.

In addition, the following parameters were monitored in accordance with the CONSORT 2010 statement for assessing the feasibility of this exploratory pilot study (Nakkawita et al., 2023; Simic et al., 2022):

1. Patient recruitment rate: specifically including the total number of potential participants contacted, the number of participants ultimately enrolled, and the primary reason for participants’ refusal to participate in the study;
2. Treatment adherence: defined as the number of participants who completed at least 80% of the total intervention regimen;
3. Protocol deviation: defined as all unexpected changes that deviated from the approved treatment protocol.

### 2.4. Data Collection

Two certified speech therapists conducted the assessment, with inter-rater agreement exceeding 0.9. Both assessors received unified training on the standardized naming battery and the scoring manual. They did not deliver the behavioral intervention, did not access each other’s prior scores, and were not informed of participants’ treatment progress unless clinically necessary. The assessment environment was a speech therapy room with ambient noise below 40 dB SPL. The phonological awareness assessment items were presented through PPT audio playback, while the naming ability assessment items were presented through PPT image playback. Both the recording and display device were a 14-inch HP laptop connected to a single-direction microphone and low-pass filter amplifier. The amplifier parameters were set to a low-pass filter of 20 Hz and a gain of 25 dB. Sound Forge 9.0 was used for voice recording and normalization, with parameters set to single channel, 16-bit quantization rate, 44.1 kHz sampling rate, and average RMS level.

### 2.5. Data Analysis

This exploratory pilot study used a single-group pre–post design with analyses stratified by aphasia type to compare outcome measures in 10 Mandarin-speaking PWAs before and after phonological awareness-based naming treatment.

The between-group independent variable was aphasia type (FA, NFA), and the within-group independent variable was the time point of measurement (pre-treatment, post-treatment). The dependent variables were the patients’ phonological awareness characteristics, naming ability, and initial consonant articulation ability before and after the treatment. The primary researcher recorded the assessment results for each outcome measure before and after treatment in real time. Two assessors jointly determined the evaluation results. All the results were stored in Excel 2019 with designated numbers for data analysis.

Statistical analyses were performed using SPSS (version 23.0). The Shapiro-Wilk test was used to assess the normality of all continuous outcome measures. Normally distributed data were expressed as mean ± standard deviation (M ± SD). Non-normally distributed data were expressed as M (P25, P75), where M indicates the median, and P25 and P75 indicate the 25th and 75th percentiles, respectively. For measures satisfying the normality assumption, a two-way mixed-design analysis of variance (ANOVA) was conducted with Group (FA vs. NFA) as the between-subject factor and Time (pre-test vs. post-test) as the within-subject factor. The main effects of Time and Group and the Time-by-Group interaction were examined, followed by simple-effects analyses where appropriate. For measures that violated the normality assumption, between-group comparisons were performed using the Mann-Whitney U test, and within-group comparisons were performed using the Wilcoxon signed-rank test. To control for the inflated Type I error risk associated with multiple testing, the Benjamini-Hochberg false discovery rate (FDR) correction was applied to all relevant families of comparisons, including main effects, interaction effects, and simple effects. All *p*-values reported were FDR-adjusted, and an adjusted *p* < 0.05 was considered statistically significant. Effect sizes were reported for all analyses: partial eta squared (η^2^) for ANOVA results, mean difference (MD) with 95% confidence interval (CI) and Cohen’s d for parametric pairwise comparisons, and *r* for non-parametric comparisons.

## 3. Results

Overall, 16 participants expressed interest in participating in the study through convenience recruitment methods, including promotional posters and information dissemination sessions. After reviewing each participant’s data, 5 did not meet the inclusion criteria (due to a history of right-hemisphere stroke, severe hearing impairment, significant blood pressure fluctuations, complete loss of oral expression ability, or using a non-Mandarin language as their primary language). The final number of participants enrolled in the study was 11. During the study implementation phase, all 10 participants completed 100% of the intervention protocol. One participant discontinued participation after completing the first two intervention sessions because they left the study site. Additionally, we observed only one deviation from the study protocol: one participant demonstrated considerable interest and compliance with the intervention. However, the total treatment duration exceeded the 15-h threshold specified in the protocol, amounting to approximately 18 h.

### 3.1. Results of the Assessment Materials’ Homogeneity

Prior to treatment, there was no significant difference in naming accuracy on Assessment 2 and Assessment 3 between the NFA group and the FA group. (t(3) = −0.95, *p* = 0.412; t(5) = −0.35, *p* = 0.741), indicating homogeneity between the training corpus and the test corpus.

### 3.2. Results of Phonological Awareness Accuracy in Mandarin-Speaking PWAs Pre- and Post-Treatment

The descriptive and significant results of parameters on phonological awareness accuracy before and after treatment are presented in Table A1. Mixed-design ANOVA revealed significant main effects of Time on all phonological awareness measures (all adjusted *p* < 0.05; partial η^2^ = 0.733–0.932). Simple-effect analyses showed significant pre-to-post improvements in both groups for rhyme awareness (FA: MD = 36.11, 95% CI = 18.04–54.18, adjusted *p* = 0.008; NFA: MD = 39.58, 95% CI = 26.88–52.28, adjusted *p* = 0.004), alliteration awareness (FA: MD = 24.60, 95% CI = 17.96–31.24, adjusted *p* < 0.001; NFA: MD = 42.86, 95% CI = 15.89–69.83, adjusted *p* = 0.030), tone awareness (FA: MD = 33.33, 95% CI = 22.62–44.04, adjusted *p* < 0.001; NFA: MD = 53.13, 95% CI = 23.29–82.96, adjusted *p* = 0.022), phoneme awareness (FA: MD = 43.75, 95% CI = 27.45 − 60.05, adjusted *p* = 0.002; NFA: MD = 41.67, 95% CI = 20.70–62.64, adjusted *p* = 0.016), syllable decoding (FA: MD = 40.28, 95% CI = 30.05–50.50, adjusted *p* < 0.001; NFA: MD = 39.59, 95% CI = 16.30–62.87, adjusted *p* = 0.024), and syllable plausibility judgments (FA: MD = 38.89, 95% CI = 12.12–65.65, adjusted *p* = 0.028; NFA: MD = 22.92, 95% CI = 14.36–31.48, adjusted *p* = 0.006). For syllable awareness, a significant improvement was found only in the FA group (MD = 41.67, 95% CI = 24.85–58.48, adjusted *p* = 0.002), whereas the improvement in the NFA group did not reach statistical significance. No significant main effect of Group or Time-by-Group interaction survived correction.

### 3.3. Results of Naming Accuracy in Mandarin-Speaking PWAs Pre- and Post-Treatment

The descriptive and significant results of parameters on naming accuracy before and after treatment are presented in Table A2. Mixed-design ANOVA revealed significant main effects of Time on naming accuracy across all three assessments (Assessment 1: adjusted *p* < 0.001, partial η^2^ = 0.802; Assessment 2: adjusted *p* < 0.001, partial η^2^ = 0.923; Assessment 3: adjusted *p* = 0.014, partial η^2^ = 0.787), indicating significant overall improvements in naming accuracy after treatment. No significant main effect of Group or Time-by-Group interaction survived correction. Simple-effect analyses showed that on Assessment 1, only the FA group demonstrated a significant improvement (MD = 8.33, 95% CI = 4.05–12.62, adjusted *p* = 0.008), whereas the improvement in the NFA group did not reach statistical significance. On Assessment 2, both groups improved significantly (FA: MD = 25.76, 95% CI = 13.08–38.44, adjusted *p* = 0.006; NFA: MD = 38.64, 95% CI = 29.30–47.98, adjusted *p* = 0.002). On Assessment 3, again only the FA group demonstrated a significant improvement (MD = 11.37, 95% CI = 6.36–16.37, adjusted *p* = 0.004), whereas the NFA group did not reach statistical significance.

### 3.4. Results of Occurrence Rate of Naming Errors in Mandarin-Speaking PWAs Pre- and Post-Treatment

The descriptive and significant results of parameters on naming error occurrence rate before and after treatment are presented in Table A3, Table A4 and Table A5.

Regarding naming error occurrence rates in Assessment 1, only two between-group baseline comparisons reached significance after correction. For E1 errors, the NFA group showed a significantly higher pre-treatment occurrence rate (Median = 20.00%, Q1–Q3 = 13.33–29.17) than the FA group (Median = 6.67%, Q1–Q3 = 5.84–7.50; U = 0.00, adjusted *p* = 0.010, *r* = −0.84). Similarly, for E6 errors, the NFA group showed a significantly higher pre-treatment rate (Median = 11.67%, Q1–Q3 = 7.50–15.84) than the FA group (Median = 3.33%, Q1–Q3 = 0.00–4.17; U = 0.50, adjusted *p* = 0.020, *r* = −0.79).

Regarding naming error occurrence rates in Assessment 2, only two comparisons reached significance after correction. At pre-test, the occurrence rate of E1 errors was significantly higher in the NFA group (Median = 15.91%, Q1–Q3 = 13.64–21.59) than in the FA group (Median = 6.82%, Q1–Q3 = 3.41–10.23; U = 1.000, adjusted *p* = 0.048, *r* = −0.755). At post-test, the occurrence rate of E10 errors was significantly higher in the FA group (Median = 9.09%, Q1–Q3 = 4.55–9.09) than in the NFA group (Median = 0.00%, Q1–Q3 = 0.00–3.41; U = 1.000, adjusted *p* = 0.038, *r* = −0.786). No other within-group, between-group, or interaction comparisons reached significance after correction.

In Assessment 3, non-parametric analyses were conducted on the occurrence rates of naming errors. After FDR correction, none of the within-group pre-to-post comparisons, between-group comparisons at pre-test or post-test, or Time-by-Group interactions reached statistical significance for any naming error type (all adjusted *p* > 0.05).

### 3.5. Results of Initial Consonant Articulation Accuracy in Mandarin-Speaking PWAs Pre- and Post-Treatment

The descriptive and significant results of initial consonant articulation accuracy before and after treatment are presented in Table A6. Mixed-design ANOVA revealed a significant main effect of Time on initial consonant articulation accuracy (MD = 21.23, 95% CI = 13.70–28.76, adjusted *p* < 0.001, partial η^2^ = 0.841), indicating significant overall improvement after treatment. Simple-effect analyses showed significant pre-to-post improvements in both the FA group (MD = 17.46, 95% CI = 7.14–27.78, adjusted *p* = 0.014) and the NFA group (MD = 25.00, 95% CI = 8.20–41.80, adjusted *p* = 0.036). No significant main effect of Group or Time-by-Group interaction survived correction.

## 4. Discussion

This exploratory pilot study investigated the feasibility and efficacy of a phonological awareness-based treatment for anomia in NFA and FA in the Mandarin context.

Overall, our findings indicate that a phonological awareness-based treatment is feasible for PWAs who present with anomia. All hospitalized participants completed the full intervention protocol. We identified only one case in which the intervention dose administered to a subject exceeded the expected value. Nevertheless, there is no evidence that this exception impacted the reporting of the overall study protocol. The primary challenge in this study was participant recruitment. The limited study duration and stringent inclusion criteria resulted in a smaller sample size. To address this limitation, further research is needed to expand the population eligible to participate in this study.

Based on phonological awareness results, patients showed significant improvement across multiple metrics after treatment. Most participants in both the FA and NFA groups showed improvements in awareness of rhyme, alliteration, syllables, tone, phonemes, syllable decoding, and judgments of syllable plausibility. These findings suggest that this training regimen exerts a potentially beneficial effect on the speech processing abilities of patients with both types of aphasia. However, awareness of syllables improved significantly only in the FA group; in the NFA group, the improvement did not reach statistical significance. Phonological awareness treatment does not directly focus on real words that pose retrieval challenges for the patient, which is a prevalent approach in traditional therapy. The fundamental reason is that Mandarin syllables are composed of initials, finals, and tones, and syllables can be combined to form words (Feng et al., 2019). In Mandarin, the syllable is a salient and functional unit in phonological processing, and syllabic structure directly constrains lexical access (Feng et al., 2019). Syllable awareness tasks, such as syllable counting, segmentation, or manipulation, typically require explicit operation on syllabic units, which imposes additional demands on phonological working memory and executive control. Because NFA often involves more pronounced motor-planning and sequencing difficulties, the NFA group may have found these metalinguistic operations more challenging, limiting their ability to benefit from this specific training component. In addition to syllable-level training, phoneme-level training aims to strengthen the multi-modal distributed representations of phonemes. This helps patients relearn the sub-components of speech and lays the foundation for word-level phonological awareness tasks. Practice with a limited set of minimal consonant phoneme pairs, core single vowels, and the four tones of Mandarin can effectively help patients initiate phonological awareness training. Additionally, nonwords help patients focus on phonology rather than word semantics (Banco et al., 2025). Previous research on PMT (Braun et al., 2025) indicates that functional communication can be improved without specifically training personally relevant real words. Although real words are introduced later in the therapeutic process and inevitably involve semantic processing, both the IA model and the PDP model illustrate the interconnection between phonology and semantics.

Furthermore, phonological awareness treatment helped participants learn to use multi-modal phonological representation domains as a self-cueing strategy during phonological processing for word retrieval. Connectionism holds that phonological representations are processed in parallel, involving several phonological representation domains (Alderete & Finley, 2023). In this study, we conceptualized the phonological representation domains for Mandarin as auditory, articulatory-motor, tactile-proprioceptive, and written Pinyin domains. Cerebral injury may partially or completely impair these domains, leading to deficits in both phonological perception and production. Nevertheless, targeted training can enhance the functionality of these domains, thus stabilizing phonological processing. The feedback and feedforward information produced by each speech event encompasses a multi-modal combination of visual, auditory, tactile, and kinesthetic elements (Kent et al., 2000). Consequently, the phonological awareness-based treatment employed various forms of auditory, tactile-proprioceptive, and visual stimulation. Audiovisual stimulation has been shown to affect phonological processing (Y. Wang et al., 2008). Because PWAs generally exhibit auditory comprehension impairments, and the visual modality allows longer stimulus presentation than the auditory modality, it produces stronger activation within the word processing system (Lorenz & Nickels, 2007). Consequently, this treatment generally prioritized the incorporation of visual stimuli during multi-modal phonological processing training, allowing patients to use their dominant domains to activate the entire phonological processing network. Research on proprioception and kinesthesia has shown that the motor and language systems draw on a common resource pool (Bose et al., 2001). Overburdening of the language system results in a shortage of available resources within the speech-motor system in PWAs with anomia. Meanwhile, dynamic cues from tactile-proprioceptive can facilitate word retrieval. Additionally, this treatment incorporated Pinyin in written form. Pinyin facilitates the refinement of the phonological awareness of Mandarin speakers at the phoneme level. Simultaneously, Pinyin reduces the cognitive load of the transition from thought to language, making the naming process more fluent (Weng, 2021). Consequently, phonological awareness treatment based on multi-modal phonological representation domains enabled PWAs to master the phonological rules of Mandarin through detection and manipulation tasks involving phonological perception and production.

Regarding the results on naming accuracy, significant improvements were observed on all three assessments in FA, which included both trained and untrained words. The theoretical basis for the generalization potential of the phonological awareness treatment is that the phonological encoding of each Mandarin word follows the same set of phonological rules, producing similar neural activation patterns during processing (Castro et al., 2022). Consequently, the treatment can improve the efficiency of phonological encoding in word retrieval (Neumann, 2018). Furthermore, intensive training targeting phonological processing improves the ability to maintain and manipulate phonological information relevant to naming (Simic et al., 2021). Notably, although the phonological awareness treatment developed in this study showed some generalization to untrained words, the results showed that the improvement rate of naming accuracy for trained words was about twice that for untrained words. This phenomenon may relate to the stability and extent of the reconstructed phonological awareness. Hebbian learning theory suggests that continuous, repetitive stimuli enhance the stability and persistence of neuronal activity (Fillingham et al., 2003). Therefore, continued practice with relevant treatment tasks on other vocabulary items could further consolidate the stability of these representations, leading to more accurate word retrieval outcomes.

In contrast, NFA showed significant improvement only on the trained words (Assessment 2). Gains on the BNT-C and on untrained words did not reach statistical significance. Several factors may account for this pattern. First, the two subtypes may differ in the locus of impairment underlying anomia. FA is often associated with deficits in phonological encoding and lexical retrieval, whereas NFA typically involves additional impairments in phonetic-motor planning and articulatory execution (Pham et al., 2025). The phonological awareness-based treatment may strengthen phonological representations and retrieval processes, thereby producing clear gains on directly trained items in both groups. However, generalization to untrained words requires flexible application of learned phonological rules to new lexical contexts. This process may be particularly demanding for the NFA group, whose phonological-motor integration is more severely compromised. Second, the test materials may also have influenced the pattern. Assessment 1 (BNT-C) includes items that were not trained and vary widely in word frequency and length. Assessment 3 consisted of untrained high-frequency nouns, so it required transfer from trained to novel phonological forms. Only Assessment 2 included the exact words used during treatment, providing direct practice effects. For the NFA group, whose phonological system may be more vulnerable, the lack of direct training on these measures may not have been sufficient to produce statistically reliable gains.

The analysis of naming errors offers some preliminary insight into the behavioral mechanisms underlying anomia in FA and NFA. However, because most error-type changes were not statistically significant, these observations should be regarded as exploratory patterns rather than as evidence that the treatment enhanced phonological–semantic interaction or modified the naming-processing system as a whole. They may therefore serve as hypotheses for future testing. In addition, this study collected no neuroimaging or electrophysiological data; thus, all error-related interpretations remain at the behavioral level within the IA model and are not intended to provide direct evidence for neural mechanisms. This behavioral approach is consistent with previous error-analysis studies in anomia (Qian et al., 2022; Minkina et al., 2016).

The baseline differences of FA and NFA in Assessment 1 and Assessment 2 are theoretically noteworthy. NFA produced significantly more omission errors than FA on both the standardized anomia tool and the treatment corpus. Omission errors are often interpreted as a failure of lexical-semantic activation to reach the phonological output stage (Dell et al., 2004; Minkina et al., 2016). Within the IA model, a higher omission rate may indicate that the target node was insufficiently activated to trigger a response. NFA may have greater difficulty delivering oral responses (Laakso & Godt, 2016), leading them to abandon their responses (omission) or provide incomplete vocal answers, which can also result in phonological errors. The higher rate of phonological errors in the NFA group is compatible with the view that NFA involves more prominent deficits in phonological encoding and articulatory programming (Pham et al., 2025).

At the post-test of Assessment 2, the occurrence rate of word formation errors was significantly higher in the FA group than in the NFA group, which possibly reflects an “over-generation” tendency at the phoneme–morpheme level. This phenomenon may be related to the “active yet imprecise” state of the speech output system following treatment. The treatment may have made the speech output of fluency-oriented patients more active. However, because the translation or integration deficits at the semantics–phonology interface were not addressed simultaneously, this active speech output is not accurately guided toward the target word form, resulting in more word formation errors.

For Assessment 3, which examined generalization to untrained words, we found no statistically detectable treatment effects. Descriptive statistics show that the occurrence rates of omission errors, semantic errors, descriptive errors, unrelated errors, and redundancy decreased in both groups after the treatment. In contrast, the occurrence rates of mixed errors and nonwords/neologisms increased after the treatment. Research based on the IA model posits that omission errors, unrelated errors, and redundancy are severe forms of naming errors (Dell et al., 2004). These errors are ascribed to a significant deficiency or disorder in the activation response of the word retrieval system. In contrast, semantic errors, phonological errors, and mixed errors are more closely tied to the target word and are classified as milder types of naming errors. The naming errors exhibited a possible trend of transition from a severe to a mild state, and this transition was consistent in both the trained and untrained word-naming tasks after treatment. Consequently, although naming accuracy for untrained words did not exhibit as significant an improvement as that of the trained words, the alteration in naming errors may also reflect a change in the word retrieval system among the patients. The decrease in the occurrence rate of severe naming errors is consistent with the possibility that the treatment may facilitate the invocation of phonological and semantic nodes, thereby changing overall activation for both the initiation and processing of word retrieval. Furthermore, the increase in mixed errors, which are both phonetically and semantically related to the target word, is consistent with enhanced interaction between phonological and semantic nodes within the IA model. The increase in neologisms/nonwords that share high phonological similarity with the target word also suggests that patients were at least partially accessing the correct phonological representation of the target. Collectively, these findings suggest that the phonologically based naming treatment may primarily influence the entire naming processing system through feedback activation from phonological nodes that subsequently influence semantic nodes.

## 5. Limitations

Several methodological limitations of the exploratory pilot study should be acknowledged. First, the absence of a control group prevents definitive attribution of the observed improvements to the intervention. Moreover, the lack of an established gold-standard behavioral treatment for anomia in aphasia, and substantial procedural differences between the present intervention and conventional control conditions (e.g., picture naming), made it difficult to design an appropriate control arm at this early stage. Accordingly, the findings should be interpreted as evidence regarding feasibility and preliminary outcome signals.

Second, we did not conduct a follow-up assessment. Because the study was conducted at a specialized referral center and most participants lived outside the local area, returning to the hospital for follow-up evaluations was not feasible. Thus, the durability of the observed gains remains unknown.

Third, the sample was heterogeneous with respect to age, time post-stroke (months), and lesion location, which complicates interpretation of the results. Although we applied inclusion criteria (e.g., time post-stroke ≥ 1 month, exclusion of severe apraxia of speech) to reduce variability, considerable heterogeneity persisted because of the consecutive recruitment strategy and the small sample size. In addition, although demographic and basic neurological information was reported, standardized quantitative data on aphasia severity and relevant comorbidities (e.g., auditory comprehension, apraxia of speech severity) were not fully available. Accordingly, the present data cannot fully characterize the sample’s clinical heterogeneity, limiting the generalizability of the findings. Furthermore, lesion volume and precise lesion characteristics were unavailable, precluding examination of their potential influence on treatment response.

Fourth, we did not assess formal treatment fidelity using checklists, independent observations, or session reviews. Although a single certified speech-language pathologist delivered a structured protocol and the authors evaluated process metrics (e.g., recruitment rate, treatment adherence, protocol deviation) in accordance with the CONSORT extension for pilot and feasibility trials, the lack of formal fidelity measurement prevents quantitative assessment of intervention consistency and adherence. Future studies should incorporate formal fidelity checks.

Future confirmatory trials should employ larger, more homogeneous samples, include follow-up assessments as a mandatory component, and, where feasible, collect detailed lesion data and adjust for age, time post-stroke, and lesion characteristics as covariates to estimate the intervention effect and its long-term maintenance more precisely.

## 6. Conclusions

In conclusion, this exploratory pilot study suggested that a phonological awareness-based intervention was associated with improvements in phonological awareness and trained-word naming in Mandarin-speaking PWAs. The gains were most consistent on trained items, whereas improvement on untrained words was significant only in FA; thus, evidence for generalization remains limited and inconclusive. Given the uncontrolled single-group design and small sample, these findings should be regarded as preliminary and interpreted with caution. Nevertheless, they tentatively support a phonological approach tailored to the structural properties of Mandarin and underscore the need for randomized controlled trials with larger, more homogeneous samples and comprehensive follow-up assessments to establish the efficacy and durability of this intervention.

## Figures and Tables

**Table 1:** Demographic information, education level, disease duration, diagnosis, lesion sites, and classification of the participants.

Participant	Sex	Age (Years)	Education Level	Disease Duration (Months)	Diagnosis	Lesion Sites	Classification
N1	F	52	JHS	1	CI	L-Multifocal CH lesions	WA
N2	F	39	COL	5	CH	THA-BG region	BA
N3	F	45	JHS	4	CH	FL; TL; PL	BA
N4	F	52	JHS	9	CH	FL; PL	BA
N5	F	69	JHS	1	CI	MCA territory	WA
N6	M	75	JHS	4	CI	MCA territory	TCSA
N7	M	42	COL	2	CH	BG, PV	WA
N8	M	56	SHS	2	CH	BG, FL, TL	WA
N9	M	71	SHS	2	CI	L-Multifocal CH lesions	WA
N10	M	38	COL	3	CH	BG	BA

N, number; M, male; F, female; COL, college; JHS, junior high school; SHS, senior high school; CI, cerebral infarction; CH, cerebral hemorrhage; L-Multifocal CH lesions, multifocal lesions in left cerebral hemisphere; THA-BG region, thalamus-basal ganglia region; FL, frontal lobe; TL, temporal lobe; PL, parietal lobe; MCA territory, middle cerebral artery territory (supplying parts of FL, TL, PL, and BG); BG, basal ganglia; PV, periventricular region; WA, Wernicke’s aphasia; BA, Broca’s aphasia; TCSA, transcortical sensory aphasia.

**Table 2:** Parallel distributed representation domains of phonology in Mandarin.

Phonological Representation Domains	Specific Description
Auditory	Listening and distinguishing the speech of the therapist and the patient based on auditory acoustic information.
Articulatory-motor	The production of speech or words, including any verbal responses such as repetition.
Tactile-proprioceptive	Noting and describing the sensation of producing speech or words, such as the vibration of the larynx and nasal alae during articulation.
Visual	Observing the positions and movements of the articulatory organs and vocalization during speech production by the therapist and patient using mirrors and mouth images.
Written-Pinyin	The written form of Pinyin, including initial consonants, finals, and tones, presented in Pinyin cards.

**Table 3:** Examples of guided questions used in the treatment.

Type of Questions	Examples
Questions that guide interpretation, exploration, and recall	What is the difference between /b/ and /p/?
	Do you remember the last time we talked about vowel training?
Questions that guide analysis	Does the vocal cord/larynx vibrate when this sound is produced?
	How do the lips move when pronouncing this sound?
	Describe the movement process of the tongue during speech?
	Are these sounds the same?
	How many syllables does this word have?
	Can you show the tone of this word with your hands?
Questions that guide analysis response (correct and incorrect)	Is the sound you say the same as the sound I say?

**Table 4:** Stimuli list (Pinyin).

Type of Stimuli	Stimuli
Impaired minimal consonant phoneme pairs	e.g., /b-p/ /d-t/
Unimpaired minimal consonant phoneme pairs	e.g., /j-q/ /g-k/
Core vowels	/a/ /i/ /u/
Tones carried by core vowels	/ā/, /á/, /ǎ/, /à/ /ī/, /í/, /ǐ/, /ì/ /ū/, /ú/, /ǔ/, /ù/
High word-likeness nonwords	mēn děi, sàng shǎ, niáng shuǎi, rào juè huǎn, huài sàn qué
Low word-likeness nonwords (with nonexistent tone combinations)	béi, huāi, kuàn rē, nuǒ mìn, fò zuán rèn, māi zěi qùn
Low word-likeness nonwords (with nonexistent phonotactic combinations)	piā, liòng, xěi hǘ, juāng cín, hià duǎi yiē, būn qò wóng
Real words	wǎn (bowl), chuáng (bed), yǎn jing (eyes), zuǐ ba (mouth), ěr duo (ears), bí zi (nose), yá shuā (toothbrush), máo jīn (towel), máo yī (sweater), yǎn jìng (glasses), lǐng dài (tie), shǒu biǎo (watch), diàn huà (telephone), tái dēng (desk lamp), zhuō zi (table), yǐ zi (chair), shā fā (sofa), kuài zi (chopsticks), sháo zi (spoon), yá gāo (toothpaste), shōu yīn jī (radio), chuī fēng jī (hair dryer)

## Data Availability

The raw data supporting the conclusions of this article will be made available by the authors on request.

## Abbreviations

The following abbreviations are used in this manuscript:

FA	Fluent aphasia
NFA	Non-fluent aphasia
IA	Interactive activation
PWA	People with aphasia
BNT	Boston Naming Test
PMT	Phonomotor treatment
PCA	Phonological component analysis
PDP	Parallel distributed processing model of phonology
BNT-C	Chinese version of the Boston Naming Test
PAAT	Phonological Awareness Assessment Tool for Mandarin-speaking people with aphasia

## Appendix A

### Appendix A.1

**Table A1:** Descriptive statistics and 2 × 2 mixed-design ANOVA with simple-effect analyses of parameters on phonological awareness accuracy aspects before the treatment (pre-test) and after the treatment (post-test) based on phonological awareness in the NFA group and FA group.

Phonological Awareness Accuracy	Group	Pre-Test (M ± SD)	Post-Test (M ± SD)	Source	*p* (raw)	*p* (adj)	Partial η^2^	Mean Diff [95% CI]	Cohen’s d [95% CI]
Awareness of rhyme (%)	FA (*n* = 6)	20.83 ± 13.69	56.94 ± 6.27						
	NFA (*n* = 4)	16.67 ± 6.81	56.25 ± 7.98						
				Time	<0.001 ***	<0.001 ***	0.892	37.85 [27.08, 48.61]	2.90 [-]
				Group	0.565	1.000	0.043		
				Time × Group	0.720	1.000	0.017		
				FA: Post-Pre	0.004 **	0.008 **		36.11 [18.04, 54.18]	2.10 [-]
				NFA: Post-Pre	0.002 **	0.004 **		39.58 [26.88, 52.28]	5.73 [-]
Awareness of alliteration (%)	FA (*n* = 6)	39.69 ± 9.36	64.29 ± 5.00						
	NFA (*n* = 4)	38.10 ± 15.06	80.95 ± 8.69						
				Time	<0.001 ***	<0.001 ***	0.911	33.73 [25.15, 42.30]	3.20 [-]
				Group	0.166	1.000	0.225		
				Time × Group	0.040 *	0.880	0.430		
				FA: Post-Pre	<0.001 ***	<0.001 ***		24.60 [17.96, 31.24]	3.89 [-]
				NFA: Post-Pre	0.015 *	<0.030 *		42.86 [15.89, 69.83]	2.92 [-]
Awareness of syllables (%)	FA (*n* = 6)	40.00 ± 17.89	81.67 ± 11.69						
	NFA (*n* = 4)	37.50 ± 17.08	77.50 ± 17.08						
				Time	<0.001 ***	0.008 **	0.813	40.83 [24.86, 56.80]	2.07 [-]
				Group	0.671	1.000	0.024		
				Time × Group	0.907	1.000	0.002		
				FA: Post-Pre	0.001 **	0.002 **		41.67 [24.85, 58.48]	2.60 [-]
				NFA: Post-Pre	0.066	0.132		40.00 [−5.01, 85.01]	1.16 [-]
Awareness of tone (%)	FA (*n* = 6)	22.92 ± 14.61	56.25 ± 6.85						
	NFA (*n* = 4)	28.13 ± 11.97	81.25 ± 7.22						
				Time	<0.001 ***	<0.001 ***	0.919	43.23 [29.06, 57.40]	3.36 [-]
				Group	0.022 *	1.000	0.499		
				Time × Group	0.060 *	1.000	0.374		
				FA: Post-Pre	<0.001 ***	<0.001 ***		33.33 [22.62, 44.04]	3.27 [-]
				NFA: Post-Pre	0.011*	0.022 *		53.13 [23.29, 82.96]	2.91 [-]
Awareness of phonemes (%)	FA (*n* = 6)	25.00 ± 12.64	68.75 ± 4.37						
	NFA (*n* = 4)	33.33 ± 18.94	75.00 ± 6.80						
				Time	<0.001 ***	0.001 **	0.910	42.71 [31.77, 53.65]	3.33 [-]
				Group	0.236	1.000	0.170		
				Time × Group	0.832	1.000	0.006		
				FA: Post-Pre	0.001 **	0.002 **		43.75 [27.45, 60.05]	2.82 [-]
				NFA: Post-Pre	0.008 **	0.016 *		41.67 [20.70, 62.64]	2.90 [-]
Syllable decoding (%)	FA (*n* = 6)	36.11 ± 9.00	76.39 ± 5.05						
	NFA (*n* = 4)	42.71 ± 21.62	82.29 ± 10.96						
				Time	<0.001 ***	<0.001 ***	0.932	39.93 [31.77, 48.73]	3.44 [-]
				Group	0.380	1.000	0.097		
				Time × Group	0.930	1.000	0.001		
				FA: Post-Pre	<0.001 ***	<0.001 ***		40.28 [30.05, 50.50]	3.22 [-]
				NFA: Post-Pre	0.012 *	0.024 *		39.59 [16.30, 62.87]	2.48 [-]
Judgments of syllable plausibility (%)	FA (*n* = 6)	36.11 ± 21.35	75.00 ± 5.27						
	NFA (*n* = 4)	56.25 ± 7.22	79.17 ± 5.89						
				Time	0.002 **	0.044 *	0.733	30.90 [15.70, 46.11]	1.55 [-]
				Group	0.045 *	1.000	0.412		
				Time × Group	0.261	1.000	0.155		
				FA: Post-Pre	0.014 *	0.028 *		38.89 [12.12, 65.65]	1.53 [-]
				NFA: Post-Pre	0.003 **	0.006 **		22.92 [14.36, 31.48]	2.96 [-]

Note. FA, fluent aphasia; NFA, non-fluent aphasia. Values are M ± SD. For the main effects and interaction, *p*(adj) is the global Bonferroni-adjusted *p*-value (raw *p* × 22), where 22 is the total number of outcome measures that were normally distributed in this study (11 pre-test and 11 post-test indicators). For simple effects, *p*(adj) is the Bonferroni-adjusted *p*-value (raw *p* × 2). Partial η^2^ = partial eta-squared. Cohen’s d for simple effects was computed from paired-samples *t*-tests; Cohen’s d for the main effect of time was converted from partial eta-squared. Owing to the small sample size, 95% confidence intervals for Cohen’s d were not estimated and are therefore marked as [-]. * *p* < 0.05; ** *p* < 0.01; *** *p* < 0.001.

### Appendix A.2

**Table A2:** Descriptive statistics and 2 × 2 mixed-design ANOVA with simple-effect analyses of parameters on naming accuracy aspects before the treatment (pre-test) and after the treatment (post-test) based on phonological awareness in the NFA group and FA group.

Naming Accuracy	Group	Pre-Test (M ± SD)	Post-Test (M ± SD)	Source	F (1, 8)/t(df)	*p* (raw)	*p* (adj)	Partial η^2^	Mean Diff [95% CI]	Cohen’s d [95% CI]
Assessment 1 (%)	FA (*n* = 6)	50.56 ± 8.54	58.89 ± 8.61							
	NFA (*n* = 4)	35.00 ± 13.47	59.17 ± 13.44							
				Time	32.433	<0.001 ***	<0.001 ***	0.802	16.25 [9.67, 22.83]	1.80 [-]
				Group	1.485	0.258	1.000	0.157		
				Time × Group	7.701	0.024 *	1.000	0.490		
				FA: Post-Pre	5.000	0.004 **	0.008 **		8.33 [4.05, 12.62]	1.36 [-]
				NFA: Post-Pre	3.596	0.037 *	0.074		24.17 [2.78, 45.56]	1.79 [-]
Assessment 2 (%)	FA (*n* = 6)	43.18 ± 17.19	68.94 ± 12.34							
	NFA (*n* = 4)	29.55 ± 15.07	68.19 ± 11.74							
				Time	95.539	<0.001 ***	<0.001 ***	0.923	32.20 [24.60, 39.80]	3.46 [-]
				Group	0.682	0.433	1.000	0.079		
				Time × Group	3.820	0.086	1.000	0.323		
				FA: Post-Pre	5.222	0.003 **	0.006 **		25.76 [13.08, 38.44]	2.13 [-]
				NFA: Post-Pre	13.168	0.001 **	0.002 **		38.64 [29.30, 47.98]	5.38 [-]
Assessment 3 (%)	FA (*n* = 6)	43.94 ± 17.64	55.30 ± 16.37							
	NFA (*n* = 4)	35.23 ± 10.08	53.41 ± 12.52							
				Time	29.504	0.001 **	0.014 *	0.787	14.77 [8.50, 21.04]	1.92 [-]
				Group	0.319	0.588	1.000	0.038		
				Time × Group	1.570	0.246	1.000	0.164		
				FA: Post-Pre	5.841	0.002 **	0.004 **		11.37 [6.36, 16.37]	2.38 [-]
				NFA: Post-Pre	2.954	0.060	0.120		18.18 [−1.40, 37.76]	1.21 [-]

Note. FA, fluent aphasia; NFA, non-fluent aphasia. Values are M ± SD. For the main effects and interaction, *p*(adj) is the global Bonferroni-adjusted *p*-value (raw *p* × 22), where 22 is the total number of outcome measures that were normally distributed in this study (11 pre-test and 11 post-test indicators). For simple effects, *p*(adj) is the Bonferroni-adjusted *p*-value (raw *p* × 2). Partial η^2^ = partial eta-squared. Cohen’s d for simple effects was computed from paired-samples *t*-tests; Cohen’s d for the main effect of time was converted from partial eta-squared. Owing to the small sample size, 95% confidence intervals for Cohen’s d were not estimated and are therefore marked as [-]. * *p* < 0.05; ** *p* < 0.01; *** *p* < 0.001.

### Appendix A.3

**Table A3:** Descriptive statistics and non-parametric analyses with simple-effect comparisons of parameters on the occurrence rate of naming errors in Assessment 1 before the treatment (pre-test) and after the treatment (post-test) based on phonological awareness in the NFA group and FA group.

Naming Errors	Group	Pre-Test M (P25, P75)	Post-Test M (P25, P75)	Source	Z/U	*p* (raw)	*p* (adj)	*r*
E1 (%)	FA (*n* = 6)	6.67 (5.84, 7.50)	3.33 (0.00, 3.33)	FA: Post-Pre	−2.232	0.031 *	0.062	−0.911
	NFA (*n* = 4)	20.00 (13.33, 29.17)	1.67 (0.00, 5.84)	NFA: Post-Pre	−1.826	0.125	0.250	−0.913
				Pre-test FA vs. NFA	0.000	0.005 **	0.010 *	−0.837
				Post-test FA vs. NFA	12.000	1.000	1.000	0.000
				Interaction (Δ FA vs. Δ NFA)	0.000	0.010 *	0.600	−0.821
E2 (%)	FA (*n* = 6)	11.67 (10.00, 17.50)	6.67 (5.84, 10.83)	FA: Post-Pre	−2.232	0.031 *	0.062	−0.911
	NFA (*n* = 4)	11.67 (7.50, 15.84)	6.67 (4.16, 9.17)	NFA: Post-Pre	−1.414	0.500	1.000	−0.707
				Pre-test FA vs. NFA	9.500	0.643	1.000	−0.175
				Post-test FA vs. NFA	10.000	0.752	1.000	−0.145
				Interaction (Δ FA vs. Δ NFA)	12.000	1.000	1.000	0.000
E3 (%)	FA (*n* = 6)	5.00 (2.50, 6.67)	3.33 (0.00, 3.33)	FA: Post-Pre	−1.890	0.125	0.250	−0.772
	NFA (*n* = 4)	5.00 (0.83, 9.17)	3.34 (0.00, 6.67)	NFA: Post-Pre	−1.414	0.500	1.000	−0.707
				Pre-test FA vs. NFA	11.000	0.995	1.000	−0.071
				Post-test FA vs. NFA	10.000	0.695	1.000	−0.144
				Interaction (Δ FA vs. Δ NFA)	7.000	0.295	1.000	−0.357
E4 (%)	FA (*n* = 6)	6.67 (2.50, 7.50)	1.67 (0.00, 3.33)	FA: Post-Pre	−2.041	0.063	0.126	−0.833
	NFA (*n* = 4)	3.33 (3.33, 5.84)	0.00 (0.00, 2.50)	NFA: Post-Pre	−1.633	0.250	0.500	−0.816
				Pre-test FA vs. NFA	8.000	0.486	0.972	−0.288
				Post-test FA vs. NFA	9.000	0.571	1.000	−0.237
				Interaction (Δ FA vs. Δ NFA)	8.500	0.586	1.000	−0.243
E5 (%)	FA (*n* = 6)	0.00 (0.00, 5.00)	8.34 (5.84, 10.00)	FA: Post-Pre	−1.903	0.094	0.188	−0.777
	NFA (*n* = 4)	1.67 (0.00, 3.33)	10.00 (7.50, 10.00)	NFA: Post-Pre	−1.890	0.125	0.250	−0.945
				Pre-test FA vs. NFA	11.000	1.000	1.000	−0.077
				Post-test FA vs. NFA	8.500	0.476	0.952	−0.270
				Interaction (Δ FA vs. Δ NFA)	10.000	0.843	1.000	−0.146
E6 (%)	FA (*n* = 6)	3.33 (0.00, 4.17)	3.33 (0.00, 6.67)	FA: Post-Pre	−0.531	0.625	1.000	−0.217
	NFA (*n* = 4)	11.67 (7.50, 15.84)	5.00 (3.33, 9.17)	NFA: Post-Pre	−1.633	0.250	0.500	−0.816
				Pre-test FA vs. NFA	0.500	0.010 *	0.020 *	−0.790
				Post-test FA vs. NFA	7.000	0.319	0.638	−0.354
				Interaction (Δ FA vs. Δ NFA)	3.000	0.048 *	1.000	−0.612
E7 (%)	FA (*n* = 6)	3.33 (3.33, 4.17)	8.34 (6.67, 10.00)	FA: Post-Pre	−2.232	0.031 *	0.062	−0.911
	NFA (*n* = 4)	3.34 (0.00, 11.67)	6.67 (6.67, 14.17)	NFA: Post-Pre	−1.473	0.250	0.500	−0.736
				Pre-test FA vs. NFA	11.500	1.000	1.000	−0.036
				Post-test FA vs. NFA	10.500	0.786	1.000	−0.116
				Interaction (Δ FA vs. Δ NFA)	8.000	0.462	1.000	−0.282
E8 (%)	FA (*n* = 6)	6.67 (6.67, 10.00)	6.67 (3.33, 7.50)	FA: Post-Pre	−1.518	0.250	0.500	−0.620
	NFA (*n* = 4)	5.00 (0.83, 6.67)	1.67 (0.00, 5.84)	NFA: Post-Pre	−1.342	0.500	1.000	−0.671
				Pre-test FA vs. NFA	4.000	0.143	0.286	−0.610
				Post-test FA vs. NFA	4.500	0.105	0.210	−0.530
				Interaction (Δ FA vs. Δ NFA)	11.000	0.914	1.000	−0.070
E9 (%)	FA (*n* = 6)	1.67 (0.00, 3.33)	0.00 (0.00, 0.83)	FA: Post-Pre	−1.414	0.500	1.000	−0.577
	NFA (*n* = 4)	0.00 (0.00,0.00)	0.00 (0.00, 2.50)	NFA: Post-Pre	−1.000	1.000	1.000	−0.500
				Pre-test FA vs. NFA	6.000	0.200	0.400	−0.507
				Post-test FA vs. NFA	11.000	1.000	1.000	−0.097
				Interaction (Δ FA vs. Δ NFA)	6.000	0.267	1.000	−0.500
E10 (%)	FA (*n* = 6)	0.00 (0.00, 3.33)	0.00 (0.00, 3.33)	FA: Post-Pre	0.000	1.000	1.000	0.000
	NFA (*n* = 4)	0.00 (0.00, 2.50)	0.00 (0.00,0.00)	NFA: Post-Pre	−1.000	1.000	1.000	−0.500
				Pre-test FA vs. NFA	11.000	1.000	1.000	−0.084
				Post-test FA vs. NFA	8.000	0.467	0.934	−0.387
				Interaction (Δ FA vs. Δ NFA)	9.500	0.633	1.000	−0.208

Note. FA = fluent aphasia (*n* = 6); NFA = non-fluent aphasia (*n* = 4). E1, omission errors; E2, semantic errors; E3, descriptive errors; E4, unrelated errors; E5, mixed errors; E6, phonological errors; E7, nonwords/neologisms; E8, redundancy; E9, visual similarity errors; E10, word formation errors. Values are presented as M (P25, P75). *p*(adj) = adjusted *p*-value. For within-group comparisons, *p*(adj) = *p* × 2. For between-group comparisons, *p*(adj) = *p* × 2. For the interaction (between-group comparison of change scores), *p*(adj) = *p* × 60 (60 = total number of non-parametric outcome measures: 30 measures × 2 time points). Effect sizes are rank-biserial correlation coefficients (*r*), calculated as *r* = Z/√N for Wilcoxon signed-rank tests and *r* = 1 − 2U/(n_1_n_2_) for Mann–Whitney U tests. All tests were two-tailed. * *p* < 0.05; ** *p* < 0.01.

### Appendix A.4

**Table A4:** Descriptive statistics and non-parametric analyses with simple-effect comparisons of parameters on the occurrence rate of naming errors in Assessment 2 before the treatment (pre-test) and after the treatment (post-test) based on phonological awareness in the NFA group and FA group.

Naming Errors	Group	Pre-Test M (P25, P75)	Post-Test M (P25, P75)	Source	Z/U	*p* (raw)	*p* (adj)	*r*
E1 (%)	FA (*n* = 6)	6.82 (3.41, 10.23)	0.00 (0.00, 0.00)	FA: Post-Pre	−2.041	0.063	0.125	−0.834
	NFA (*n* = 4)	15.91 (13.64, 21.59)	0.00 (0.00, 0.00)	NFA: Post-Pre	−1.841	0.125	0.250	−0.921
				Pre-test FA vs. NFA	1.000	0.024 *	0.048 *	−0.755
				Post-test FA vs. NFA	12.000	1.000	1.000	0.000
				Interaction (Δ FA vs. Δ NFA)	1.000	0.024 *	1.000	−0.755
E2 (%)	FA (*n* = 6)	9.09 (7.96, 18.18)	2.28 (0.00, 5.69)	FA: Post-Pre	−2.023	0.063	0.125	−0.826
	NFA (*n* = 4)	9.09 (5.69, 12.50)	0.00 (0.00, 6.82)	NFA: Post-Pre	−1.857	0.125	0.250	−0.929
				Pre-test FA vs. NFA	9.500	0.629	1.000	−0.181
				Post-test FA vs. NFA	10.000	0.929	1.000	−0.153
				Interaction (Δ FA vs. Δ NFA)	12.000	1.000	1.000	0.000
E3 (%)	FA (*n* = 6)	4.55 (3.41, 4.55)	2.28 (0.00, 5.69)	FA: Post-Pre	−1.089	0.500	1.000	−0.445
	NFA (*n* = 4)	9.09 (5.6, 12.50)	4.55 (4.55, 4.55)	NFA: Post-Pre	−1.633	0.250	0.500	−0.817
				Pre-test FA vs. NFA	2.500	0.029	0.058	−0.724
				Post-test FA vs. NFA	8.000	0.414	0.828	−0.309
				Interaction (Δ FA vs. Δ NFA)	7.500	0.376	1.000	−0.315
E4 (%)	FA (*n* = 6)	6.82 (4.55, 9.09)	0.00 (0.00, 0.00)	FA: Post-Pre	−2.251	0.031 *	0.062	−0.919
	NFA (*n* = 4)	4.55 (4.55, 7.96)	0.00 (0.00, 3.41)	NFA: Post-Pre	−1.633	0.250	0.500	−0.817
				Pre-test FA vs. NFA	9.000	0.571	1.000	−0.237
				Post-test FA vs. NFA	9.000	0.400	0.800	−0.387
				Interaction (Δ FA vs. Δ NFA)	7.500	0.381	1.000	−0.336
E5 (%)	FA (*n* = 6)	0.00 (0.00, 5.69)	4.55 (0.00, 9.09)	FA: Post-Pre	−1.342	0.500	1.000	−0.548
	NFA (*n* = 4)	4.55 (4.55, 4.55)	6.82 (4.55, 12.50)	NFA: Post-Pre	−1.342	0.500	1.000	−0.671
				Pre-test FA vs. NFA	6.000	0.171	0.342	−0.447
				Post-test FA vs. NFA	7.000	0.319	0.638	−0.354
				Interaction (Δ FA vs. Δ NFA)	10.500	0.929	1.000	−0.114
E6 (%)	FA (*n* = 6)	4.55 (0.00, 5.69)	2.28 (0.00, 5.69)	FA: Post-Pre	−0.276	1.000	1.000	−0.113
	NFA (*n* = 4)	9.09 (5.69, 9.09)	6.82 (4.55, 9.09)	NFA: Post-Pre	−0.577	1.000	1.000	−0.289
				Pre-test FA vs. NFA	4.000	0.110	0.220	−0.577
				Post-test FA vs. NFA	5.000	0.238	0.476	−0.500
				Interaction (Δ FA vs. Δ NFA)	10.500	0.814	1.000	−0.102
E7 (%)	FA (*n* = 6)	4.55 (4.55, 10.23)	9.09 (6.82, 9.09)	FA: Post-Pre	−0.414	0.625	1.000	−0.169
	NFA (*n* = 4)	9.09 (2.27, 9.09)	9.09 (5.69, 9.09)	NFA: Post-Pre	−1.000	1.000	1.000	−0.500
				Pre-test FA vs. NFA	10.500	0.752	1.000	−0.108
				Post-test FA vs. NFA	11.500	1.000	1.000	−0.048
				Interaction (Δ FA vs. Δ NFA)	10.500	0.814	1.000	−0.106
E8 (%)	FA (*n* = 6)	9.09 (9.09, 13.64)	2.28 (0.00, 4.55)	FA: Post-Pre	−2.201	0.031 *	0.062	−0.898
	NFA (*n* = 4)	6.82 (1.14, 9.09)	0.00 (0.00, 0.00)	NFA: Post-Pre	−1.633	0.250	0.500	−0.817
				Pre-test FA vs. NFA	4.000	0.143	0.286	−0.610
				Post-test FA vs. NFA	6.000	0.200	0.400	−0.507
				Interaction (Δ FA vs. Δ NFA)	9.000	0.619	1.000	−0.217
E9 (%)	FA (*n* = 6)	2.28 (0.00, 4.55)	0.00 (0.00, 0.00)	FA: Post-Pre	−1.826	0.125	0.250	−0.746
	NFA (*n* = 4)	0.00 (0.00, 0.00)	0.00 (0.00, 0.00)	NFA: Post-Pre	0.000	1.000	1.000	0.000
				Pre-test FA vs. NFA	6.000	0.200	0.400	−0.507
				Post-test FA vs. NFA	12.000	1.000	1.000	0.000
				Interaction (Δ FA vs. Δ NFA)	6.000	0.200	1.000	−0.507
E10 (%)	FA (*n* = 6)	2.28 (0.00, 4.55)	9.09 (4.55, 9.09)	FA: Post-Pre	−2.041	0.063	0.125	−0.834
	NFA (*n* = 4)	4.55 (4.55, 4.55)	0.00 (0.00, 3.41)	NFA: Post-Pre	−1.732	0.250	0.500	−0.866
				Pre-test FA vs. NFA	6.000	0.200	0.400	−0.507
				Post-test FA vs. NFA	1.000	0.019 *	0.038 *	−0.786
				Interaction (Δ FA vs. Δ NFA)	0.000	0.005 **	1	−0.832

Note. FA = fluent aphasia (*n* = 6); NFA = non-fluent aphasia (*n* = 4). E1, omission errors; E2, semantic errors; E3, descriptive errors; E4, unrelated errors; E5, mixed errors; E6, phonological errors; E7, nonwords/neologisms; E8, redundancy; E9, visual similarity errors; E10, word formation errors. Values are presented as M (P25, P75). *p*(adj) = adjusted *p*-value. For within-group comparisons, *p*(adj) = *p* × 2. For between-group comparisons, *p*(adj) = *p* × 2. For the interaction (between-group comparison of change scores), *p*(adj) = *p* × 60 (60 = total number of non-parametric outcome measures: 30 measures × 2 time points). Effect sizes are rank-biserial correlation coefficients (*r*), calculated as *r* = Z/√N for Wilcoxon signed-rank tests and *r* = 1 − 2U/(n_1_n_2_) for Mann–Whitney U tests. All tests were two-tailed. * *p* < 0.05; ** *p* < 0.01.

### Appendix A.5

**Table A5:** Descriptive statistics and non-parametric analyses with simple-effect comparisons of parameters on the occurrence rate of naming errors in Assessment 3 before the treatment (pre-test) and after the treatment (post-test) based on phonological awareness in the NFA group and FA group.

Naming Errors	Group	Pre-Test M (P25, P75)	Post-Test M (P25, P75)	Source	Z/U	*p* (raw)	*p* (adj)	*r*
E1 (%)	FA (*n* = 6)	4.55 (3.41, 9.09)	0.00 (0.00, 4.55)	FA: Post-Pre	−2.070	0.063	0.125	−0.845
	NFA (*n* = 4)	11.37 (9.09, 13.64)	2.28 (0.00, 4.55)	NFA: Post-Pre	−1.841	0.125	0.250	−0.921
				Pre-test FA vs. NFA	2.000	0.033 *	0.066	−0.707
				Post-test FA vs. NFA	10.000	1.000	1.000	−0.158
				Interaction (Δ FA vs. Δ NFA)	4.000	0.105	1.000	−0.555
E2 (%)	FA (*n* = 6)	13.64 (7.96, 18.18)	6.82 (0.00, 10.23)	FA: Post-Pre	−2.032	0.063	0.125	−0.829
	NFA (*n* = 4)	6.82 (4.55, 9.09)	4.55 (4.55, 7.96)	NFA: Post-Pre	−0.577	1.000	1.000	−0.289
				Pre-test FA vs. NFA	5.000	0.238	0.476	−0.500
				Post-test FA vs. NFA	11.500	0.886	1.000	−0.035
				Interaction (Δ FA vs. Δ NFA)	4.500	0.138	1.000	−0.523
E3 (%)	FA (*n* = 6)	4.55 (0.00, 9.09)	4.55 (0.00, 4.55)	FA: Post-Pre	−1.414	0.500	1.000	−0.577
	NFA (*n* = 4)	11.37 (2.27, 13.64)	2.28 (0.00, 4.55)	NFA: Post-Pre	−1.604	0.250	0.500	−0.802
				Pre-test FA vs. NFA	6.000	0.290	0.580	−0.417
				Post-test FA vs. NFA	10.000	1.000	1.000	−0.158
				Interaction (Δ FA vs. Δ NFA)	5.000	0.119	1.000	−0.510
E4 (%)	FA (*n* = 6)	6.82 (3.41, 10.23)	2.28 (0.00, 5.69)	FA: Post-Pre	−1.604	0.250	0.500	−0.655
	NFA (*n* = 4)	4.55 (1.14, 7.96)	2.28 (0.00, 4.55)	NFA: Post-Pre	−1.000	1.000	1.000	−0.500
				Pre-test FA vs. NFA	8.500	0.524	1.000	−0.248
				Post-test FA vs. NFA	11.000	1.000	1.000	−0.075
				Interaction (Δ FA vs. Δ NFA)	9.500	0.619	1.000	−0.190
E5 (%)	FA (*n* = 6)	0.00 (0.00, 1.14)	9.09 (6.82, 13.64)	FA: Post-Pre	−2.041	0.063	0.125	−0.833
	NFA (*n* = 4)	4.55 (1.14, 4.55)	11.37 (2.27, 13.64)	NFA: Post-Pre	−1.512	0.250	0.500	−0.756
				Pre-test FA vs. NFA	5.000	0.190	0.380	−0.553
				Post-test FA vs. NFA	11.000	0.924	1.000	−0.072
				Interaction (Δ FA vs. Δ NFA)	9.000	0.486	1.000	−0.217
E6 (%)	FA (*n* = 6)	4.55 (0.00, 5.69)	4.55 (3.41, 9.09)	FA: Post-Pre	−0.816	0.750	1.000	−0.333
	NFA (*n* = 4)	9.09 (9.09, 9.09)	9.09 (5.69, 12.50)	NFA: Post-Pre	−0.447	1.000	1.000	−0.224
				Pre-test FA vs. NFA	2.000	0.038 *	0.076	−0.732
				Post-test FA vs. NFA	5.500	0.276	0.552	−0.467
				Interaction (Δ FA vs. Δ NFA)	10.500	0.871	1.000	−0.108
E7 (%)	FA (*n* = 6)	6.82 (4.55, 9.09)	9.09 (7.96, 10.23)	FA: Post-Pre	−1.633	0.250	0.500	−0.667
	NFA (*n* = 4)	11.37 (2.27, 13.64)	11.37 (9.09, 13.64)	NFA: Post-Pre	−1.000	1.000	1.000	−0.500
				Pre-test FA vs. NFA	7.500	0.338	0.676	−0.318
				Post-test FA vs. NFA	7.000	0.343	0.686	−0.386
				Interaction (Δ FA vs. Δ NFA)	10.500	0.929	1.000	−0.114
E8 (%)	FA (*n* = 6)	9.09 (9.09, 13.64)	0.00 (0.00, 4.55)	FA: Post-Pre	−2.264	0.031 *	0.062	−0.924
	NFA (*n* = 4)	6.82 (1.14, 9.09)	0.00 (0.00, 6.82)	NFA: Post-Pre	−1.342	0.500	1.000	−0.671
				Pre-test FA vs. NFA	4.000	0.143	0.286	−0.610
				Post-test FA vs. NFA	12.000	1.000	1.000	0.000
				Interaction (Δ FA vs. Δ NFA)	4.000	0.100	1.000	−0.577
E9 (%)	FA (*n* = 6)	2.28 (0.00, 4.55)	0.00 (0.00, 4.55)	FA: Post-Pre	−0.577	1.000	1.000	−0.235
	NFA (*n* = 4)	0.00 (0.00, 0.00)	0.00 (0.00, 6.82)	NFA: Post-Pre	−1.000	1.000	1.000	−0.500
				Pre-test FA vs. NFA	6.000	0.200	0.400	−0.507
				Post-test FA vs. NFA	11.000	1.000	1.000	−0.084
				Interaction (Δ FA vs. Δ NFA)	8.000	0.638	1.000	−0.306
E10 (%)	FA (*n* = 6)	2.28 (0.00, 5.69)	4.55 (0.00, 9.09)	FA: Post-Pre	−1.134	0.500	1.000	−0.463
	NFA (*n* = 4)	4.55 (4.55, 7.96)	0.00 (0.00, 3.42)	NFA: Post-Pre	−1.890	0.125	0.250	−0.945
				Pre-test FA vs. NFA	6.500	0.319	0.638	−0.403
				Post-test FA vs. NFA	6.000	0.262	0.524	−0.439
				Interaction (Δ FA vs. Δ NFA)	0.500	0.019 *	1.000	−0.792

Note. FA = fluent aphasia (*n* = 6); NFA = non-fluent aphasia (*n* = 4). E1, omission errors; E2, semantic errors; E3, descriptive errors; E4, unrelated errors; E5, mixed errors; E6, phonological errors; E7, nonwords/neologisms; E8, redundancy; E9, visual similarity errors; E10, word formation errors. Values are presented as M (P25, P75). *p*(adj) = adjusted *p*-value. For within-group comparisons, *p*(adj) = *p* × 2. For between-group comparisons, *p*(adj) = *p* × 2. For the interaction (between-group comparison of change scores), *p*(adj) = *p* × 60 (60 = total number of non-parametric outcome measures: 30 measures × 2 time points). Effect sizes are rank-biserial correlation coefficients (*r*), calculated as *r* = Z/√N for Wilcoxon signed-rank tests and *r* = 1 − 2U/(n_1_n_2_) for Mann–Whitney U tests. All tests were two-tailed. * *p* < 0.05.

### Appendix A.6

**Table A6:** Descriptive statistics and 2 × 2 mixed-design ANOVA with simple-effect analyses of parameters on articulation accuracy aspects before the treatment (pre-test) and after the treatment (post-test) based on phonological awareness in the NFA group and FA group.

Variable	Group	Pre-Test (M ± SD)	Post-Test (M ± SD)	Source	F (1, 8)/t(df)	*p* (raw)	*p* (adj)	Partial η^2^	Mean Diff [95% CI]	Cohen’s d [95% CI]
Articulation accuracy (%)	FA (*n* = 6)	63.49 ± 17.21	80.95 ± 8.52							
	NFA (*n* = 4)	60.71 ± 19.20	85.72 ± 12.30							
				Time	42.309	<0.001 ***	<0.001 ***	0.841	21.23 [13.70, 28.76]	1.97 [-]
				Group	0.013	0.913	1.000	0.002		
				Time × Group	1.335	0.281	1.000	0.143		
				FA: Post-Pre	4.348	0.007 **	0.014 *		17.46 [7.14, 27.78]	1.78 [-]
				NFA: Post-Pre	4.736	0.018 *	0.036 *		25.00 [8.20, 41.80]	1.93 [-]

Note. FA, fluent aphasia; NFA, non-fluent aphasia. Values are M ± SD. For the main effects and interaction, *p*(adj) is the global Bonferroni-adjusted *p*-value (raw *p* × 22), where 22 is the total number of outcome measures that were normally distributed in this study (11 pre-test and 11 post-test indicators). For simple effects, *p*(adj) is the Bonferroni-adjusted *p*-value (raw *p* × 2). Partial η^2^ = partial eta-squared. Cohen’s d for simple effects was computed from paired-samples *t*-tests; Cohen’s d for the main effect of time was converted from partial eta-squared. Owing to the small sample size, 95% confidence intervals for Cohen’s d were not estimated and are therefore marked as [-]. * *p* < 0.05; ** *p* < 0.01; *** *p* < 0.001.
